# New Insights in the Removal of the Hydantoins, Oxidation Product of Pyrimidines, via the Base Excision and Nucleotide Incision Repair Pathways

**DOI:** 10.1371/journal.pone.0021039

**Published:** 2011-07-25

**Authors:** Modesto Redrejo-Rodríguez, Christine Saint-Pierre, Sophie Couve, Abdelghani Mazouzi, Alexander A. Ishchenko, Didier Gasparutto, Murat Saparbaev

**Affiliations:** 1 Groupe Réparation de l'ADN, CNRS UMR8200, Université Paris-Sud, Institut de Cancérologie Gustave Roussy, Villejuif, France; 2 Laboratoire Lésions des Acides Nucléiques, SCIB/UMR E3 CEA-UJF, INAC, CEA, Grenoble, France; University of Massachusetts, United States of America

## Abstract

**Background:**

Oxidative damage to DNA, if not repaired, can be both miscoding and blocking. These genetic alterations can lead to mutations and/or cell death, which in turn cause cancer and aging. Oxidized DNA bases are substrates for two overlapping repair pathways: base excision (BER) and nucleotide incision repair (NIR). Hydantoin derivatives such as 5-hydroxyhydantoin (5OH-Hyd) and 5-methyl-5-hydroxyhydantoin (5OH-5Me-Hyd), major products of cytosine and thymine oxidative degradation pathways, respectively, have been detected in cancer cells and ancient DNA. Hydantoins are blocking lesions for DNA polymerases and excised by bacterial and yeast DNA glycosylases in the BER pathway. However little is known about repair of pyrimidine-derived hydantoins in human cells.

**Methodology/Principal Findings:**

Here, using both denaturing PAGE and MALDI-TOF MS analyses we report that the bacterial, yeast and human AP endonucleases can incise duplex DNA 5′ next to 5OH-Hyd and 5OH-5Me-Hyd thus initiating the NIR pathway. We have fully reconstituted the NIR pathway for these lesions *in vitro* using purified human proteins. Depletion of Nfo in *E. coli* and APE1 in HeLa cells abolishes the NIR activity in cell-free extracts. Importantly, a number of redundant DNA glycosylase activities can excise hydantoin residues, including human NTH1, NEIL1 and NEIL2 and the former protein being a major DNA glycosylase activity in HeLa cells extracts.

**Conclusions/Significance:**

This study demonstrates that both BER and NIR pathways can compete and/or back-up each other to remove hydantoin DNA lesions *in vivo*.

## Introduction

Endogenous aerobic metabolism and variety of exogenous factors generate reactive oxygen species (ROS), which can damage macromolecules including lipids, proteins and nucleic acids. DNA has a limited chemical stability and it is one of the most biologically critical targets for ROS, with more than 80 base modifications identified so far [1]. Oxidative damage to DNA can induce mutations that cause cancer, cell death or senescence. In Archaea, Bacteria and Eukarya, as well as in some large DNA viruses, oxidatively damaged DNA bases are removed in two repair pathways: base excision repair (BER) and nucleotide incision repair (NIR) [2], [3], [4], [5], [6]. In the classical BER pathway, a DNA glycosylase excises the base, leaving as an end product either an apurinic/apyrimidinic (AP) site or a single-stranded DNA break with 3′-sugar phosphate groups which must be removed prior the gap-filling synthesis step [7], [8]. Alternatively, in the NIR pathway, an AP endonuclease makes an incision 5′ next to a damaged base in a DNA glycosylase-independent manner, providing a proper 3′-OH group for DNA polymerization and a 5′-dangling damaged nucleotide [3]. Although the majority of oxidized DNA bases are removed in the BER pathway initiated by multiple DNA glycosylases [9], [10] certain types of oxidative DNA damage such as the alpha-anomeric 2′-deoxynucleosides (αdA, αdT and αdC) cannot be repaired by DNA glycosylases/AP lyases but rather by the AP endonucleases in the alternative NIR pathway [11], [12], [13]. Furthermore, oxidatively damaged pyrimidines including 5,6-dihydrothymine (DHT), 5,6-dihydrouracil (DHU), 5-hydroxyuracil (5OHU) and 5-hydroxycytosine (5OHC) are substrates for the both BER and NIR pathways suggesting that latter pathway can serve as back-up system to counteract oxidative stress [3], [13], [14].

In human cells the major AP endonuclease 1, APE1/Ref-1/HAP-1, initiates NIR pathway by cleaving duplex DNA 5′ next to oxidatively damaged bases [13]. In the past, APE1 was independently discovered as an abasic site-specific endonuclease homologous to the *E. coli* Xth protein [15] and as a redox-regulator of the DNA binding domain of Fos-Jun, Jun-Jun, AP-1 proteins and several other transcription factors [16]. In addition to AP endonuclease and NIR activities, APE1 exhibits other DNA repair activities: 3′→5′ exonuclease, 3′-phosphodiesterase, 3′-phosphatase and RNase H [15]. Although pH, ionic strength and divalent cation requirements of the APE1-catalyzed NIR versus AP endonuclease are dramatically different, we have demonstrated that the intracellular environment of human cells can support NIR function [17]. The APE1 repair activities are divalent metal ion dependent, indeed, structural studies have shown that APE1 contain metal-binding site(s) that can held several metal ions including Sm^+3^, Pb^+2^, Ca^+2^, Mn^+2^ and Mg^+2^ [18], [19]. Previously, we have demonstrated that Zn^+2^ cations can support APE1-catalyzed NIR activity in human cell-free extracts [13]. Interestingly, zinc is the second most abundant transition metal in the body after iron [20] therefore it could play biological role in stimulating APE1-NIR activity *in vivo*.

**Figure 1:**
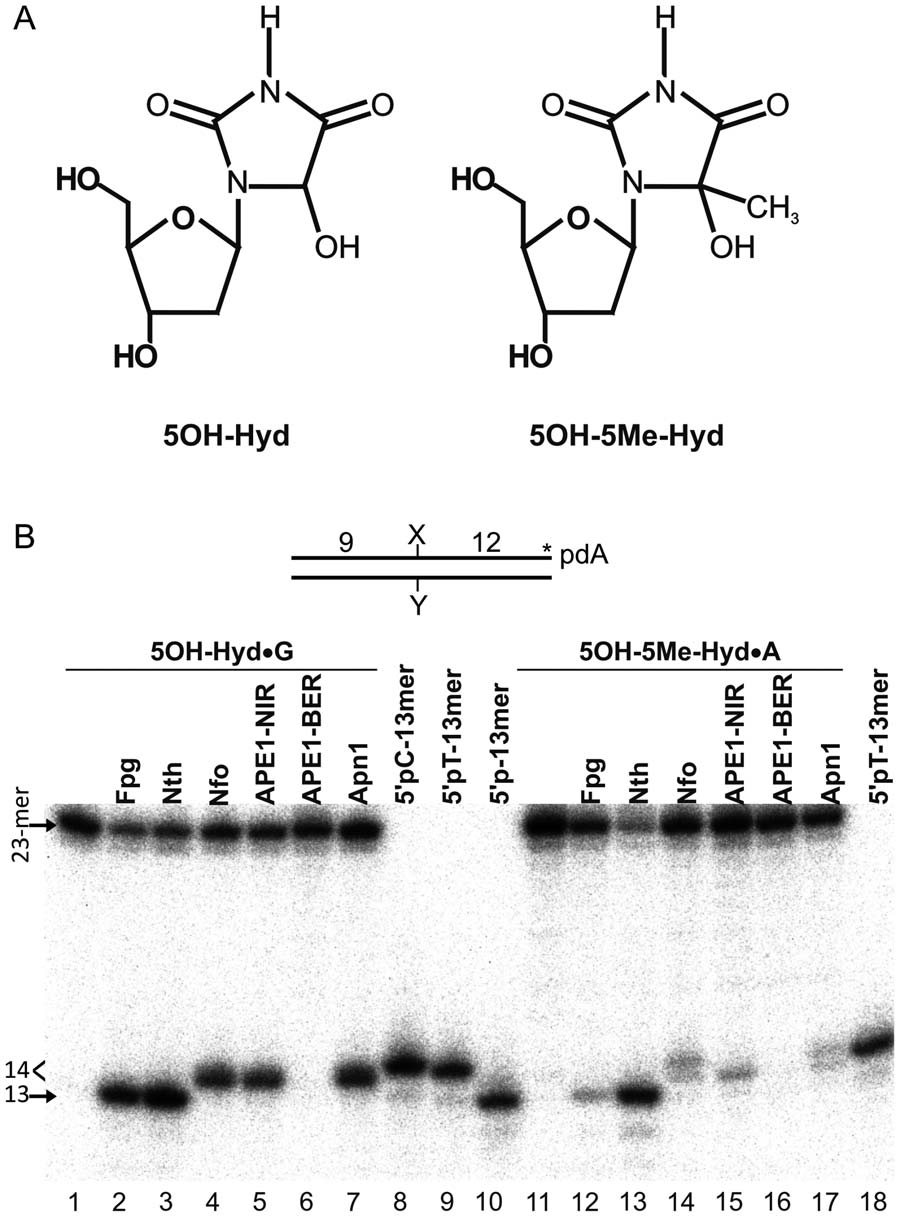
Pyrimidine hydantoins are substrates for the NIR and BER pathways. (**A**) Chemical structures of 5-hydroxyhydantoin-2′-deoxynucleoside (5OH-dHyd) and 5-hydroxy-5-methylhydantoin-2′-deoxynucleoside (5OH-5Me-dHyd). (**B**) Denaturing PAGE analysis of the cleavage products after incubation of the 3′-[^32^P]-labelled 5OH-Hyd•G (lanes 1–7) and 5OH-5Me-Hyd•A (lanes 11–18) duplex oligonucleotides with the DNA glycosylases/AP lyases (10 nM) and AP endonucleases (0.5 nM) of different origins. Lanes 1 and 11, control no enzyme; lanes 4 and 14, Nfo; lanes 5 and 14, APE1 under NIR conditions; lanes 6 and 15, APE1 under BER conditions; lanes 7 and 17, Apn1; lanes 2 and 12, Fpg; lanes 3 and 13, Nth; size markers: lane 8, 14-mer fragment with 5′-terminal pdC nucleotide; lanes 9 and 18, 14-mer fragment with 5′-terminal pT nucleotide; lane 10, 13-mer fragment with 5′-phosphate. For details see Materials and Methods. The arrows and “<” symbol denote the position of the 23-mer, 13-mer and 14-mer fragments, respectively.

Initial oxidation of pyrimidines predominantly occurs at C_5_-C_6_ double bond, generating thymine and uracil glycols, 5,6-dihydro-5,6-dihydroxythymine and 5,6-dihydroxy-5,6-dihydro-uracil, respectively. Hydantoin, also known as glycolyurea, derivatives: 5-hydroxyhydantoin (5OH-Hyd) and 5-hydroxy-5-methylhydantoin (5OH-5Me-Hyd) (Figure 1A) have been shown to be major oxidative products formed by exposure of pyrimidine derivatives to several oxidizing agents including radiation-induced •OH radical [21], [22], [23], [24], [25]. Biological relevance of these lesions is supported by their presence in tumour cells [26], [27] and also in cancer patients under radiotherapy [28]. Also, pyrimidine-derived hydantoins have been found in ancient DNA [29]. 5OH-Hyd and 5OH-5Me-Hyd can act as potential blocking lesions for DNA polymerases, suggesting their cytotoxic effect if not repaired *in vivo* [30]. Recent works have shown that translesional synthesis (TLS) DNA polymerases can efficiently by-pass hydantoin lesions, albeit with low fidelity rates [31], [32]. The studies of the molecular mechanisms of repair of these genotoxic lesions have biological implications since they could provide new tools for accurate amplification of ancient DNA [33].

Previously, it has been shown that *E. coli* Nth, Nei and Fpg as well as *S. cerevisiae* Ntg1 and 2 can initiate the BER pathway for 5OH-Hyd and 5OH-5Me-Hyd residues in DNA [34], [35]. Here, we demonstrate that the AP endonucleases of *E. coli* Nfo, yeast Apn1 and human APE1 initiate the NIR pathway by incising the duplex DNA containing 5OH-Hyd and 5OH-5Me-Hyd residues. Using Matrix Assisted Laser Desorption Ionisation Time-Of-Flight (MALDI-TOF) Mass Spectrometry (MS) analysis we show that the AP endonucleases cleave 5′ next to the pyrimidine-derived hydantoin lesions when present in the duplex DNA substrate. The removal of 5OH-Hyd and 5OH-5Me-Hyd residues in the both BER and NIR pathways in bacteria, yeast and human cells has been characterized. A number of redundant DNA glycosylase activities excising the pyrimidine-derived hydantoins have been identified, including human NTH1, NEIL1 and NEIL2 DNA glycosylases. The roles of BER and NIR, as back-up complementary pathways for oxidized DNA bases *in vivo*, are discussed.

## Results

### Pyrimidine hydantoins are substrates for the NIR pathway

*E. coli* and *S. cerevisiae* bifunctional DNA glycosylases initiate BER pathway by excising 5OH-Hyd and 5OH-5Me-Hyd residues when present in duplex DNA [34]. To examine whether these oxidized bases can be also substrates of the AP endonucleases involved in the NIR pathway we incubated the 3′-[^32^P]-labelled 5OH-Hyd•G and 5OH-5Me-Hyd•A substrates with the *E. coli* Nfo, *S. cerevisiae* Apn1 and human APE1 proteins. As shown in Figure 1B, all three AP endonucleases tested cleave the sugar phosphate backbone 5′ next to 5OH-Hyd nucleotide, generating ∼14-mer (n+1) fragment that migrates slower (lanes 4,5 and 7) than 13-mer (n) DNA glycosylases cleavage product (lanes 2 and 3). In agreement with our previous observations, APE1 efficiently incise 5OH-Hyd•G under NIR conditions, whereas no activity was detected under BER conditions (lanes 5 *versus* 6) [13]. Unexpectedly, the (n+1) AP endonuclease-cleavage fragments migrate faster than the 3′-[^32^P]-labelled 14-mer size marker oligonucleotides containing either cytosine or thymine at 5′ end (lanes 4,5,7 *versus* 9,10), suggesting that either cleavage fragments with 5′-terminal nucleotide migrate faster as compared to 14-mer size marker or that 5OH-Hyd may undergo some transformation after enzymatic cleavage.

Interestingly, all AP endonucleases tested also incised 5OH-5Me-Hyd•A, albeit with much lower efficiency as compared to 5OH-Hyd•G duplex (lanes 14,15,17 *versus* 4,5,7). Again, APE1 cleaved 5OH-5Me-Hyd•A only under NIR conditions generating the cleavage product with the same size as with 5OH-Hyd•G oligonucleotide (lanes 15 and 5). In contrast, Nfo and Apn1-catalyzed incision resulted in two closely migrating (n+1) fragments (lanes 14 and 17), upper-band co-migrates with 14-mer size marker (lane 18) and lower-band co-migrates with 5OH-Hyd•G NIR-cleavage fragment (lanes 4,5,7). Taken together, these results suggest that the Apn1 and Nfo-generated upper-band corresponds to the fragment containing a 5′-dangling 5OH-5Me-Hyd nucleotide whereas the lower-band is similar to that observed with 5OH-Hyd•G substrate and may correspond to fragment containing a decomposed form of 5OH-5Me-Hyd. We may propose that bigger molecular weight difference between 5OH-5Me-Hyd and its putative degradation product resulted in a better electrophoretic resolution on the gel as compared to 5OH-Hyd and its corresponding putative degradation product. Importantly, Nfo-catalyzed cleavage of 5OH-5Me-Hyd•A under NIR condition gives essentially the same proportion of two closely migrated bands on the gel as compared to the Nfos' standard reaction condition. Furthermore, longer incubations and/or simultaneous treatment of 5OH-5Me-Hyd•A with Nfo and APE1 do not change the migration pattern of cleavage products (Supporting Information Figure S1). The difference between APE1 and other AP endonucleases observed on 5OH-5Me-Hyd•A substrate may imply difference in the mechanism of action of these enzymes. Since, oxidative stress can generate 5OH-5Me-Hyd in DNA from both thymine and 5-methylcytosine residues, we investigated base-pair specificity of DNA repair enzymes using two duplexes 5OH-5Me-Hyd•A or 5OH-5Me-Hyd•G as substrates. APE1, Nfo, Apn1 and Nth incise 5OH-5Me-Hyd•A and 5OH-5Me-Hyd•G with the same efficiency (data not shown).

### Study of the mechanism of action of DNA repair enzymes on the pyrimidine-derived hydantoins by MALDI-TOF mass spectrometry

**Figure 2:**
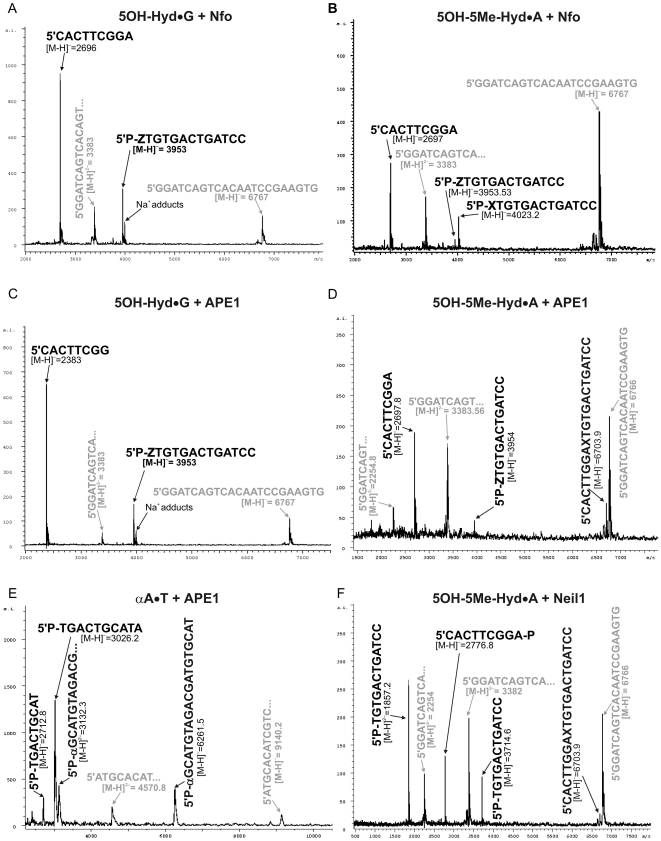
MALDI-TOF MS analysis of the mixture of oligonucleotides arising from the incubation of the 22-mer DNA duplexes containing hydantoin residues with AP endonucleases. Typically, 40 pmol of the lesion containing oligonucleotide duplexes were incubated with either 3 units of Nfo or 170 ng of APE1 or 100 ng of NEIL1 in the appropriate reaction buffer (10 µL) at 37°C for 30 min. The products were desalted on a MicroSpin G-25 column, prior subjection to the MALDI-TOF MS measurements. (**A**) Treatment of 5OH-Hyd•G duplex with Nfo. (**B**) Treatment of 5OH-5Me-Hyd•A duplex with Nfo. (**C**) Treatment of 5OH-Hyd•G duplex with APE1. (**D**) Treatment of 5OH-5Me-Hyd•A duplex with APE1. (**E**) Treatment of αdA•T duplex with APE1. (**F**) Treatment of 5OH-5Me-Hyd•A duplex with NEIL1. Peaks corresponding to complementary strand are indicated in grey. The sequence of complementary strand corresponding to a double charged species ([M-H]^2−^ = 3383) is shown in truncated form due to space limitation.

Previously, the mechanism of action of the AP endonucleases on oxidatively damaged bases was studied by analysing the migration pattern of 3′-end labelled cleavage DNA fragments in denaturing PAGE [3]. Here, for the first time we performed MALDI-TOF MS analysis of the reaction products of the AP endonucleases and DNA glycosylases when acting on 5OH-Hyd and 5OH-5Me-Hyd lesions in duplex DNA. Analysis of the mass spectrum of the reaction products resulting from the incision of 5OH-Hyd•G by Nfo showed two mono-charged cleavage product peaks: one at [M-H]^−^ = 2696 Da corresponding to the 9-mer oligonucleotide released 5′ upstream to the lesion 5′-CACTTCGGA ([M-H]^−^ with calculated mass 2698 Da), and the other one with molecular mass [M-H]^−^ = 3953 Da corresponding to a 13-mer oligonucleotide 5′-p-ZTGTGACTGATCC (Figure 2A). While, the expected Nfo-cleavage fragment 5′-p-XTGTGACTGATCC released 3′ downstream to the lesion should have a calculated mass [M-H]^−^ = 4009.5 Da. The difference of 56.5 Da between expected mass and experimental value suggest that 5OH-Hyd residue (X of 116 Da) is decomposed to an ureido residue (Z of 59 Da). In addition to the cleavage products we can see the presence of the mono- and bi-charged peaks corresponding to the complementary strand ([M-H]^−^ and [M-2H]^2−^, respectively). Importantly, the respective amounts of cleavage fragments containing ureido and 5OH-Hyd varied among the experiments. As shown in Figure 2B, the spectrum of products after incubation of 5OH-5Me-Hyd with Nfo contains a peak corresponding to the 13-mer 5′-p-XTGTGACTGATCC with [M-H]^−^ = 4023 Da which closely corresponds to calculated [M-H]^−^ = 4023.5 Da and an additional peak with [M-H]^−^ = 3953.5 Da corresponding to the 13-mer 5′-p-ZTGTGACTGATCC that contains an ureido residue. As expected the MALDI-TOF MS analysis did not reveal any cleavage of the complementary strands (Figure 2).

Interestingly, MALDI-TOF MS analysis showed that APE1-catalyzed incision of 5OH-Hyd•G and 5OH-5Me-Hyd•A gave rise only to ureido-containing fragment and no hydantoin-containing cleavage products were observed (Figure 2C and D). In addition, APE1 extend the nick to the gap by 3′→5′ exonuclease activity generating shorter 8 mer downstream cleavage fragment 5′-CACTTCGG ([M-H]^−^ with calculated mass 2383 Da) (Figure 2C). It should be stressed that the control MALDI-TOF MS analysis of the newly synthesized hydantoin-containing oligonucleotides prior to incubation with DNA repair enzymes confirmed the integrity of 5OH-Hyd and 5OH-5Me-Hyd nucleotides and did not reveal any trace of degradation products such as ureido adducts [30], [36]. Therefore, this data strongly suggest that ureido adducts occur during and/or after the AP endonuclease-catalyzed incision. Conversion of pyrimidine-derived hydantoins to ureido during NIR raises the question whether the AP endonucleases could affect stability of a damaged nucleotide during incision step. For this we examined reaction products of the APE1-catalyzed incision of 30-mer αdA•T oligonucleotide duplex by MALDI-TOF MS. As expected, we found two mono-charged peaks one at [M-H]^−^ = 3026.5 Da corresponding to the 10-mer oligonucleotide released 5′ upstream to αdA nucleotide 5′-pTGACTGCATA (calculated [M-H]^−^ = 3026 Da and the other one at [M-H]^−^ = 6261.5 Da corresponding to the 20-mer oligonucleotide released 3′ downstream to the lesion 5′-p-αdA-pGCATGTAGACGATGTGCAT (calculated [M-H]^−^ = 6260 Da (Figure 2E). This result indicates that APE1 cleaves duplex DNA 5′ next to αdA further confirming the mechanism of action of the AP endonuclease. Importantly, analysis of mass spectrum did not reveal any chemical modifications of αdA residue in the cleavage products suggesting that the AP endonucleases do not degrade 5′-dangling αdA residues.

Next we employed MALDI-TOF MS analysis to investigate the mechanism of action of NEIL1, a human DNA glycosylase that excises pyrimidine hydantoins residues in the BER pathway. Similar to *E. coli* Fpg and Nei proteins, NEIL1 is a bi-functional DNA glycosylase endowed with an AP lyase activity that incises DNA at abasic sites by a β,δ-elimination mechanism and leaves single-strand DNA break carrying a phosphate residue at the 3′ and 5′-termini (Bandaru et al., 2002). As expected, MALDI-TOF MS analysis revealed two mono-charged peaks one at [M-H]^−^ = 2776.8 Da corresponding to the 9-mer oligonucleotide released 5′ upstream to the lesion 5′-CACTTCGGAp (calculated [M-H]^−^ = 2778) and the other one at [M-H]^−^ = 3714.6 Da corresponding to the 12-mer oligonucleotide released 3′ downstream to the lesion 5′-pTGTGACTGATCC (calculated [M-H]^−^ = 3715.5) (Figure 2F). This result corroborates with previous data obtained using denaturing PAGE separation technique (Figure 1B).

### Activity of the AP endonucleases on oligonucleotide duplexes containing thymine glycol and urea residues

Thymine glycol is a major nucleobase lesion that may be formed within DNA by several oxidative processes. This oxidized form of pyrimidine residue, as hydantoins residues, exhibit a ring-chain tautomerism at C6-N1 or C5-N1 bond that may lead to degradation towards fragmented products (such as ureido or formamido residues), spontaneously and/or upon oxidative and alkali conditions [37], [38], [39]. Therefore, we examined whether Tg is a substrate for the NIR pathway. For this we used Tg-containing oligonucleotide 34-mer, 30-mer and 19-mer duplexes Tg-34•A, Tg-30•A and 19Tg-IW•A, respectively as substrates for wild-type (WT) APE1 and NIR-deficient APE1K98E mutant. Denaturing PAGE analysis revealed that the both APE1 proteins incise 5′-[^32^P]-labelled Tg-34•A and Tg-30•A oligonucleotide duplexes but not 19Tg-IW•A duplex (Supporting Information Figure S2A). Interestingly, Tg-34 and Tg-30 oligonucleotides migrated as a double band suggesting the presence of degradation product of Tg. Indeed, the slow migrating “upper-band” cannot be incised by wild-type APE1 and NIR-deficient APE1-K98E mutant, whereas the fast migrating “lower-band” band was incised by both APE1. The MS analysis of the “lower-band” fragment purified form the gel showed that it contains an ureido nucleotide (data not shown) indicating that the degradation product of Tg but not intact Tg residue is a substrate for APE1. The urea-containing oligonucleotides purified from Tg-34•A and Tg-30•A were incised by the *E. coli* exonuclease III (Xth) protein [40] and also by the NIR-deficient APE1 (D308 and K98A/R185A) mutants (Supporting Information Figure S2A,B) whereas 5OH-Hyd•G and 5OH-5Me-Hyd•A duplexes were not suggesting that the later duplexes do not contain urea (Supporting Information Figures S1). Furthermore, APE1 can incise urea-containing DNA substrate under both NIR and BER conditions with similar efficiency suggesting that urea residue closely mimics an abasic site and can be recognized by APE1 under both reaction conditions (Supporting Information Figure S2A). Importantly, APE1-catalyzed incision of 5OH-Hyd•G and 5OH-5Me-Hyd•A oligonucleotide duplexes is strongly inhibited under BER condition thus ruling out a possible presence of ureido degradation products in our oligonucleotide preparations (Figure 1B). Taken together these data suggest that although Tg is not a substrate for APE1, its degradation products can be repaired in the NIR pathway.

### Activity of various DNA glycosylases on oligonucleotide duplexes containing 5OH-Hyd and 5OH-5Me-Hyd lesions

**Figure 3:**
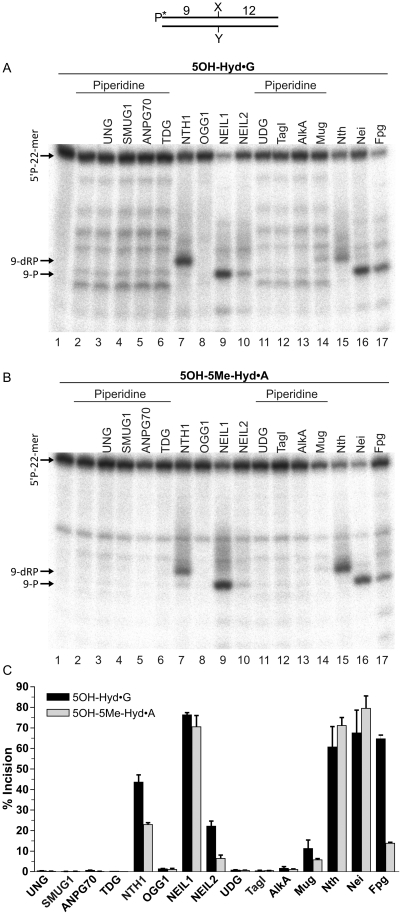
Activity of various *E. coli* and human DNA glycosylases on pyrimidine-derived hydantoins. 5 nM of 5′-[^32^P]-labelled oligonucleotide duplexes were incubated with 50 nM of DNA glycosylase for 30 min at 37°C. For the mono-functional DNA glycosylases a light piperidine treatment was performed to reveal potential AP sites. (**A**) 5OH-Hyd•G oligonucleotide duplex; (**B**) 5OH-5Me-Hyd•A oligonucleotide duplex; (**C**) Graphic representation of the means of enzymatic activities from three independent experiments. The background values representing control oligonucleotides degradation in absence of enzyme (treated or not with piperidine) were subtracted. For details see Materials and Methods.

Previous studies have demonstrated that pyrimidine-derived hydantoins are substrates for bi-functional DNA glycosylases in *E. coli* and yeast. Here, we investigated whether 5OH-Hyd and 5OH-5Me-Hyd residues are also substrates for the previously characterized bacterial and human DNA glycosylases. As shown in Figure 3, the 5′-[^32^P]-labeled 5OH-Hyd•G and 5OH-5Me-Hyd•A oligonucleotide duplexes were challenged with a variety of highly purified DNA glycosylases. Since not all DNA glycosylases possess AP site-nicking activity, the samples, after incubation with the mono-functional DNA glycosylases, were treated with light piperidine treatment [10% (v/v) piperidine at 37°C for 45 min]. in order to cleave DNA at the potential abasic sites generated by the excision of the modified base. Control light piperidine treatment of 5OH-Hyd•G duplex resulted in slight defragmentation of the oligonucleotide to short DNA fragments indicating the presence of alkaline labile sites most likely due to spontaneous depurination (Figure 3A, lane 2). Nevertheless, 5OH-Hyd and 5OH-5Me-Hyd residues were resistant to piperidine treatment and no cleavage at the lesion site was observed in the absence of DNA glycosylase treatment (Figure 3A,B lane 2). In agreement with our previous observations, *E. coli* Fpg, Nth and Nei excise with good efficiency both hydantoins (Figure 3A,B lanes 15–17). As expected NTH1 and NEIL1, human homologues of Nth and Nei, respectively, excise with comparable efficiency both 5OH-Hyd and 5OH-5Me-Hyd residues (lanes 7 and 9). While, NEIL2 a human paralogue of NEIL1 shows only weak activity towards hydantoins (lane 10). Despite being used in 10-fold molar excess, none of the mono-functional DNA glycosylases used: UNG, SMUG1, ANPG70, TDG, UDG, TagI, AlkA were able to excise the hydantoins. Except *E. coli* MUG, Mismatch Uracil DNA Glycosylase, which showed weak activity on both 5OH-Hyd•G and 5OH-5Me-Hyd•A substrates (lane 14). Interestingly, TDG, a human homologue of MUG, does not show any detectable activity on the hydantoins (lane 6). Overall, the *E. coli* Nei and Nth proteins show a slight preference for 5OH-5Me-Hyd than 5OH-Hyd (Figure 3B lanes 15–16 *vs* 3A, lanes 15–16), whereas human NEIL1 and to more extent NEIL2, NTH1 and *E. coli* Fpg excise preferentially 5OH-Hyd than 5OH-5Me-Hyd residues (Figure 3A lanes 7, 9, 10 and 17 vs 3B, lanes 7, 9, 10 and 17).

### Kinetic parameters of the incision of oligonucleotide duplexes with a single hydantoin residue by various AP endonucleases and DNA glycosylases

To examine the relative efficiency of NIR and BER pathways for the removal of 5OH-Hyd and 5OH-5Me-Hyd residues, we measured the amount of cleaved oligonucleotide as a function of the Nfo, Apn1, APE1, NTH1 and NEIL1 protein concentrations (Supporting Information Figure S3). 5OH-Hyd•G was the preferred substrate as compared to 5OH-5Me-Hyd•A for both AP endonucleases and DNA glycosylases. 5OH-5Me-Hyd•A oligonucleotide duplex was the less preferred substrate for all AP endonucleases tested, and human APE1 was the less efficient among other AP endonucleases, quickly reaching the plateau at 1–2 nM protein and incising at most 50% of 5OH-Hyd•G and 5% of 5OH-5Me-Hyd•A (Supporting Information Figure S3B). In contrast to AP endonucleases, DNA glycosylases NEIL1 and NTH1 can efficiently excise both hydantoin residues, although they also showed slight preference to 5OH-Hyd•G (Supporting Information Figure S3D). Based on these assessment studies we have adjusted the DNA repair enzyme concentration and length of incubation to cleave no more than 30% of the substrate, thereby maintaining initial velocity conditions. Then we further characterized substrate specificity of the AP endonucleases and DNA glycosylases by measuring K_M_, k_cat_ and k_cat_/K_M_ values for incision activity using 5OH-Hyd•G and 5OH-5Me-Hyd•A substrates. As shown in Table 1, the *k*_cat_/*K*_M_ values for 5OH-Hyd•G incision of all three AP endonucleases tested are the same order of magnitude as compared to that of *E. coli* Nei DNA glycosylase and 20 or 2.5 fold higher as compared to that of human DNA glycosylases NEIL1 and NTH1, respectively. The higher kinetic efficiency of the AP endonucleases towards 5OH-Hyd•G as compared to human DNA glycosylases suggests that the NIR pathway could be a major pathway for the removal of 5OH-Hyd residues *in vivo*. In contrast to Nfo, the eukaryotic AP endonucleases incise 5OH-5Me-Hyd•A substrate with very low efficiency as compared to human DNA glycosylases suggesting that 5OH-5Me-Hyd is removed mainly in the BER pathway in eukaryotic cells. Interestingly, among all DNA repair enzymes tested *E. coli* Nei and human NTH1 DNA glycosylases have the highest *k*_cat_/*K*_M_ values for cleavage of 5OH-5Me-Hyd•A.

**Table 1:** Steady-state kinetic parameters of the AP endonucleases and DNA glycosylases activities on hydantoin residues when present in duplex DNA.

DNA substrate	*5OH-Hyd•G*			*5OH-5Me-Hyd*•*A*		
Protein	K_M_ (nM)	*k*_cat_ (min^−1^)	*k*_cat_/*K*_M_ (min^−1^ µM^−1^)	*K*_M_ (nM)	*k*_cat_ (min^−1^)	*k*_cat_/*K*_M_ (min^−1^ µM^−1^)
Nei	4.8±1.5	2.5±0.14	520	5.85±0.95	4.7±0.15	803
Nfo	17.8±3	2.7±0.18	150	30.3±6.6	1.8±0.2	59.5
Apn1	30.3±5.9	4.8±0.45	158		>0.4	
APE1	115±34.5	13.3±2.4	115		>0.023	
NEIL1	55.8±17	0.3±0.04	6.7	55.2±15	0.18±0.01	3.3
NTH1	36.7±8.7	1.7±0.15	50	9.6±3.2	0.7±0.05	72.7

### *In vitro* reconstitution of the human NIR pathway for 5OH-Hyd residues

**Figure 4:**
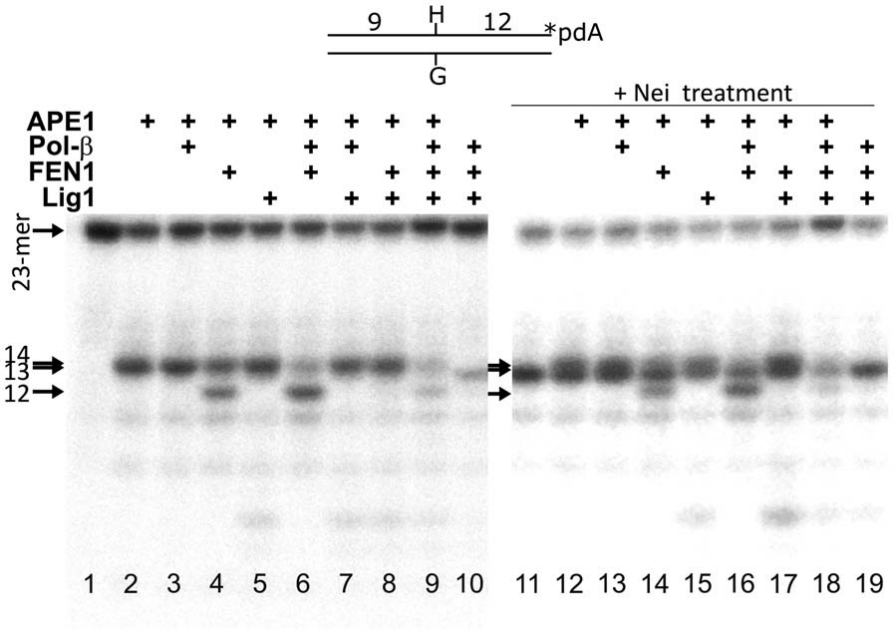
*In vitro* reconstitution of the long-patch NIR pathway using 5-OH-Hyd•G duplex DNA substrate. 3′-[α-^32^P]-ddATP-labelled 5OH-Hyd•G oligonucleotide duplex was incubated with human proteins in the reaction buffer for 1 h at 37°C. Lanes 1–10, reconstitution reactions in the presence of indicated proteins; lanes 11–20, same as 1–10 but treated with Nei. The arrows denote the position of the 23-mer, 14-mer, 13-mer and 12-mer fragments. Note that “23-mer” denotes the position of both the 3′-[^32^P]-labelled 5OH-Hyd•G substrate and repaired C•G oligonucleotide duplex. For details see Materials and Methods.

Recently, we have reconstituted *in vitro* the human NIR pathway for αdA•T oligonucleotide duplex using four purified proteins APE1, flap endonuclease 1 (FEN1), DNA polymerase β (POLβ) and DNA ligase I (LIG1) [41]. As described previously, for the NIR reconstitution assay we used 2 mM ATP in addition to 3 mM MgCl_2_ which allowed us to bring the concentration of free Mg^2+^ down to 1 mM, due to binding of magnesium cations by ATP. Here, we examined whether 5OH-Hyd residues can be efficiently removed in the APE1-initiated NIR pathway *in vitro* in DNA glycosylase-independent manner (Figure 4). Under the reaction condition used (3 mM MgCl_2_ and 2 mM ATP) which allows both NIR endonuclease activity and DNA repair synthesis, APE1 incised about 60% of 3′-[^32^P]-end labelled 5OH-Hyd•G substrate resulting in a 14-mer cleavage product (lane 2). Addition of FEN1 initiates strong 5′→3′ exonuclease degradation of 14-mer fragment (lane 4). Previously, we observed futile repair of αdA•T in the presence of APE1 and LIG1 proteins [41]. However, when APE1 cleaved 5OH-Hyd•G oligonucleotide duplex, addition of LIG1 did not restore the 23-mer full-sized fragment yet it efficiently blocked FEN1 exonuclease (lane 7). These results indicate that APE1-induced single-strand DNA breaks next to hydantoin residues would persist if the 5′-terminal dangling base is not removed. In the absence of a DNA ligase, addition of POLβ further stimulates FEN1 exonuclease activity (lane 6). Finally, in the presence of all four proteins APE1, POLβ, FEN1 and LIG1, we observed the completion of repair reaction resulting in nearly full restoration of the 23-mer fragment (lane 9). In order to verify the removal of 5OH-Hyd residues following incubation of 5OH-Hyd•G with the purified human proteins, we treated the repaired 23-mer DNA products with Nei DNA glycosylase which can incise with the high efficiency 5OH-Hyd-containing DNA (Figure 3) [34]. The appearance of a 13-mer cleavage DNA fragment after the NIR reconstitution assay will indicate the presence of 5OH-Hyd in the repaired 23-mer fragment. As expected, in the absence of APE1, no repair reactions took place (lanes 10 and 20). Importantly, Nei-treatment of the 23-mer fragment from lane 9 which was previously incubated with all four human DNA repair proteins revealed very little amount of a 13-mer cleavage product (lane 19) indicating that the absolute majority of 5OH-Hyd residues are efficiently eliminated from the 23-mer duplex during NIR reconstitution assay. These results indicate that APE1 incises 5′ next to 5OH-Hyd and allows POLβ to initiate DNA strand-displacement synthesis generating a flap-structure which is in turn cleaved by FEN1 to remove 5′-dangling hydantoin nucleotide and generate single-strand break which is then sealed by LIG1. These data strongly suggest that, under the reaction condition which enables nucleotide incision activity, DNA polymerase synthesis and ligation, 5OH-Hyd residues can be efficiently removed in the APE1-initiated NIR pathway resulting in the restoration of the DNA sequence integrity.

### DNA repair activities on 5OH-Hyd and 5OH-5Me-Hyd containing DNA in *E. coli* and human cell-free extracts

**Figure 5:**
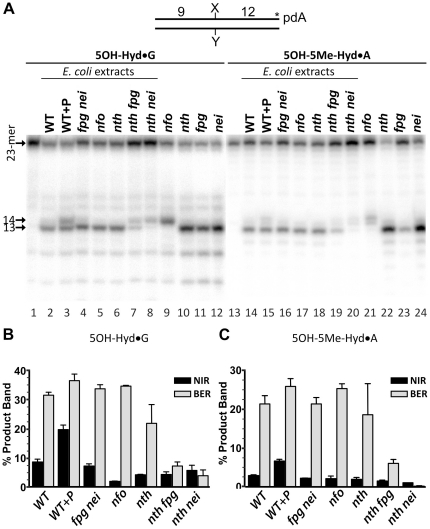
DNA repair activities towards pyrimidine-derived hydantoins in *E. coli* cell-free extracts. 3′-[α-^32^P]-ddATP-labelled oligonucleotide duplexes were incubated with either 3 µg of cell-free extract or limited amount of a purified protein in the standard DNA glycosylase reaction “BER+EDTA” buffer for 30 min at 37°C. (**A**) Denaturing PAGE analysis of the reaction products. Lane 1, control 5OH-Hyd•G with no enzyme; lanes 2–8, 5OH-Hyd•G incubated with extracts; lanes 9–12, 5OH-Hyd•G incubated with the purified proteins; lane 13, control 5OH-5Me-Hyd•A with no enzyme; lanes 14–20, 5OH-5Me-Hyd•A incubated with extracts; lanes 21–24, 5OH-5Me-Hyd•A incubated with the purified proteins. (**B, C**) Graphic representation of the mean values of DNA repair activities on 5OH-Hyd•G and 5OH-5Me-Hyd•A. DNA glycosylase (BER) and AP endonuclease-catalyzed (NIR) incisions were calculated by measuring amount of 13-mer and 14-mer products, respectively. The background values representing control oligonucleotides degradation in absence of enzyme in lanes 1 and 13 were subtracted. “**WT+P**” indicates that the expression of Nfo was induced by the exposure of cell culture to 0.25 mg/mL of paraquat. For details see Materials and Methods.

Data obtained with the purified DNA repair proteins show the redundancy of the BER and NIR pathways for the removal of pyrimidine-derived hydantoin residues in DNA. Therefore, to ascertain the respective role of these two pathways *in vivo*, we examined AP endonuclease and DNA glycosylase activities in cell-free extracts from *E. coli* and human cells. To distinguish NIR and BER activities in the extracts we used 3′-[^32^P]-labelled 5OH-Hyd•G and 5OH-5Me-Hyd•A oligonucleotide duplexes as substrates. When using these substrates the DNA glycosylases/AP lyases generate a 13-mer cleavage fragment (Figure 5A, lanes 10–12 and 22–24), whereas AP endonucleases generate a 14-mer fragment (lanes 9 and 21).

As shown in Figure 5, we detected mainly DNA glycosylase activities and very little Nfo activity on both hydantoins in the extracts from WT *E. coli* strain (lanes 2 and 14). As expected from the known induction of the Nfo protein by paraquat, addition of this oxidizing agent to growing cultures of *E. coli* increased the amount of the 14-mer fragment (lanes 3 and 15). Also, extracts from *E. coli nfo* mutant completely lack NIR activity on both DNA substrates (lanes 5 and 17) indicating that the NIR pathway in *E. coli* is absolutely dependent on the Nfo gene product. Interestingly, extracts from the *E. coli nth fpg* and *nth nei* double mutants exhibited dramatic decrease in incision activities on both 5OH-Hyd•G and 5OH-5Me-Hyd•A substrates (lanes 7, 8 and 19, 20) as compared to the extracts from *E. coli WT*, single *nth*, *nfo* and double *fpg nei* mutants (lanes 2–6 and 14–18). Importantly, in the extract from *E. coli nth nei* double mutant we detected only NIR activity and no DNA glycosylase (lanes 8 and 20) suggesting that Nth and Nei are major DNA glycosylases that remove 5OH-Hyd and 5OH-5Me-Hyd residues in the BER pathway. Taken together, these results suggest that in *E. coli* pyrimidine-derived hydantoins are mainly removed in DNA glycosylase-initiated BER pathway. However, in the absence of DNA glycosylases, the NIR pathway can serve as back-up system.

Interestingly, some reduction in the percentage of NIR cleavage products can be seen in DNA glycosylase-deficient strains: decrease from 8.6% in WT strain to 3.6% in *nth* mutant on 5OH-Hyd•G (Figure 5B) and from 2.8% in WT strain to 1% in *nth nei* double mutant on 5OH-5Me-Hyd•A (Figure 5C) suggesting that the NIR activity in *E. coli* cell-free extracts may require the presence of DNA glycosylases. To examine whether the NIR pathway functions independently of DNA glycosylase-catalyzed BER we measured the NIR and BER activities in DNA glycosylase-deficient mutants treated with paraquat. As expected, in all extracts from DNA glycosylase-deficient mutants the Nfo-catalyzed NIR activity towards 5OH-Hyd•G and 5OH-5Me-Hyd•A was induced by paraquat up to the level observed in WT strain (Supporting Information Figure S4). Based on these results we suggest that the NIR pathway functions independently of BER and does not require the presence of DNA glycosylases to remove pyrimidine-derived hydantoins.

**Figure 6:**
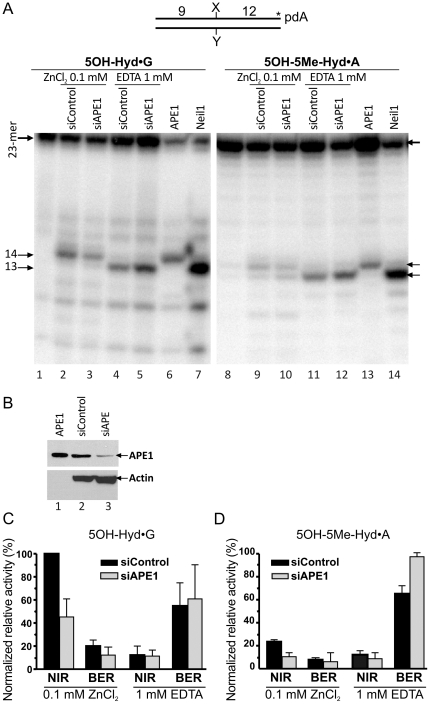
APE1-catalyzed nucleotide incision activity towards pyrimidine-derived hydantoins in HeLa cells extracts. 3′-[α-^32^P]-ddATP-labelled oligonucleotide duplexes were incubated with either 0.5 µg of HeLa cells extract or a purified protein either under “NIR+Zn^2+^” (lanes 2–3, 6 and 9–10, 13) or under “BER+EDTA” conditions (lanes 4–5, 7 and 11–12, 14) for 1 h at 37°C. (**A**) Denaturing PAGE analysis of the reaction products. Lane 1, control non-treated 5OH-Hyd•G; lanes 2 and 4, 5OH-Hyd•G incubated with extracts from HeLa cells treated with the non-specific siRNA (100 nM); lanes 3 and 5, 5OH-Hyd•G incubated with extract from HeLa cells treated with the APE1-specific siRNA (100 nM); lane 6, 5OH-Hyd•G treated with 1 nM APE1; lane 7, 5OH-Hyd•G treated with 5 nM NEIL1; lanes 8–14, same as 1–7 but with 5OH-5Me-Hyd•A as a substrate. (**B**) Western blot analysis of siRNA-induced down-regulation of the APE1 expression in HeLa cells. (**C**) Graphic representation of the mean values of DNA glycosylase (BER) and AP endonuclease (NIR) activities in extracts, representing amounts of the 13-mer and 14-mer products, respectively. The cleavage activities in each cell free extract were normalized to the relative densitometry values of actin bands on western blot in panel B. For details see Materials and Methods.

Previously, we have established that, in human cell-free extracts, the APE1-catalyzed NIR is the major activity on 5,6-dihydrouracil (DHU) and 5-hydroxycytosine (5OHC) containing DNA duplexes under NIR condition (in the presence of 0.1 mM ZnCl_2_) [13], [14]. Therefore, to assess the role of NIR pathway in the removal of 5OH-Hyd and 5OH-5Me-Hyd residues in DNA, we examined incision activities in the extracts from HeLa cells under both NIR+Zn^2+^ and BER+EDTA (in the presence of 1 mM EDTA and absence of Zn^2+^ cations that may inhibit the DNA glycosylases) conditions (Figure 6A–C). Interestingly, under the NIR+Zn^2+^ condition, we observed mainly NIR activity on the 3′-[^32^P]-labelled 5OH-Hyd•G and 5OH-5Me-Hyd•A substrates and very little DNA glycosylase-dependent cleavage (Figure 6A, lanes 2 and 9). Whereas, under the BER+EDTA condition we observed predominantly DNA glycosylase incision and no or very little NIR activity was detected (lanes 4 and 11). These results imply that the AP endonuclease and DNA glycosylase activities detected in human cell-free extracts vary dramatically upon the reaction conditions used. As expected, the APE1 silencing strongly reduces the NIR activity on 5OH-Hyd•G and 5OH-5Me-Hyd•A duplexes (lanes 3 and 10), indicating that APE1 is required for the NIR activities on hydantoins in HeLa cell-free extracts. Interestingly, down-regulation of the APE1 protein results in a dramatic increase of the DNA glycosylase activities on both DNA substrates (lanes 5 and 13). This may suggest that either the APE1 protein inhibits DNA glycosylase activities or that the transcription silencing of APE1 gene induces expression of DNA glycosylases in HeLa cells.

**Figure 7:**
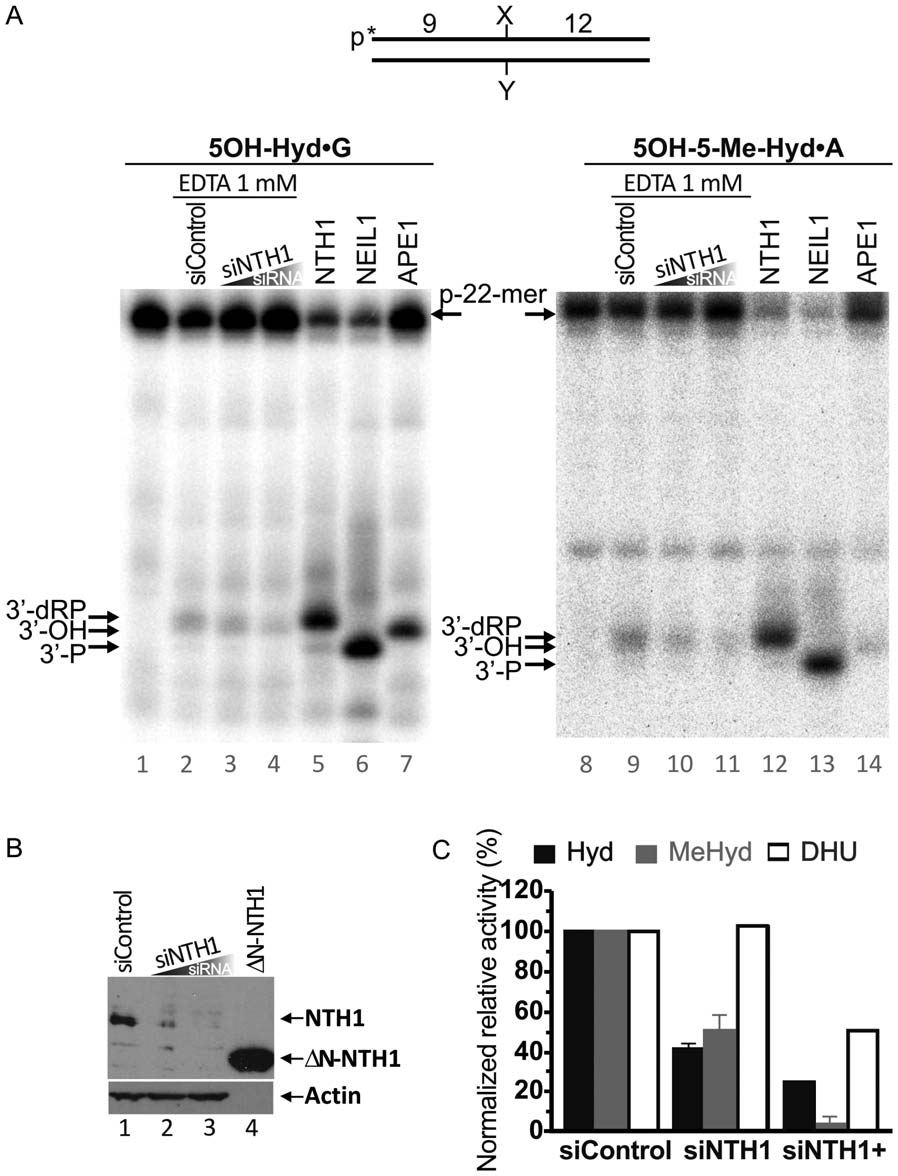
NTH1-catalyzed DNA glycosylase activity towards pyrimidine-derived hydantoins in HeLa cell extracts. 5′-[^32^P]-labelled oligonucleotide duplexes were incubated with HeLa cell extracts under “BER+EDTA” condition. (**A**) Denaturing PAGE analysis of the reaction products. Lane 1, control non-treated 5OH-Hyd•G; lane 2, as 1 but incubated with extract from HeLa cells treated with 400 nM of the non-specific siRNA; lane 3, as 1 but incubated with the extract from HeLa cells treated with 100 nM NTH1-specific siRNA; lane 4, as 3 but using 400 nM of NTH1-specific siRNA; lane 5, as 1 but with 10 nM NTH1; lane 6, as 1 but with 5 nM NEIL1; lane 7, as 1 but with 1 nM APE1; lanes 8–14, same as 1–7 but with 5OH-5Me-Hyd•A as a substrate. (**B**) Western blot analysis of the siRNA-induced down-regulation of NTH1 expression in HeLa cells. Lane 1, control HeLa cells tansfected with 400 nM of the non-specific siRNA; lane 2, HeLa cells tansfected with 100 nM of NTH1-specific siRNA; lane 3, same as 2 but 400 nM siRNA; lane 4, the purified truncated recombinant ΔN-NTH1 protein. (**C**) Graphic representation of the mean values of DNA repair activities on 5OH-Hyd•G and 5OH-5Me-Hyd•A in extracts. For comparison DNA repair activities on DHU•G substrate were also shown. The cleavage activities in each cell-free extract were normalized to the relative densitometry values of the actin bands on the western blot in panel B. The arrows denote the position of the 9-mer cleavage fragments containing 3′-dRP residue (9-dRP), 3′-hydroxyl group (3′-OH) and 3′-phosphate residue (3′-P), generated by NTH1, APE1 and NEIL1, respectively. For details see Materials and Methods.

Biochemical data demonstrate that both purified human DNA glycosylases NTH1 and NEIL1 can efficiently repair pyrimidine-derived hydantoins *in vitro* however neither NEIL1, nor NEIL2 specific activities were detected after incubation of 5′-[^32^P]-labelled 5OH-Hyd•G and 5OH-5Me-Hyd•A oligonucleotide duplexes in HeLa cells extracts under the BER+EDTA condition (Figure 7A, lanes 2 and 9). It should be noted that NEIL1-catalyzed cleavage fragments migrate faster in denaturing PAGE as compared to fragments generated by NTH1 and APE1 because they contain 3′-phosphate (3′-P) residues (Figure 7A, lanes 6 and 13 *versus* 5, 7, 12 and 14). This suggests that either 3′-P residues are removed by a 3′-phosphatase activity or that NTH1 could be a major hydantoin-DNA glycosylase activity in human cell extracts. To test this hypothesis we down-regulated NTH1 expression using specific siRNA duplex [42]. Two concentrations of the siRNA duplex were used to down-regulate the NTH1 protein level in HeLa cells (Figure 7B). Depletion of NTH1 significantly reduces BER activity on both 5OH-Hyd•G and 5OH-5Me-Hyd•A substrates (Figure 7A, lanes 3–4 and 10–11) as compared to the control non-silenced cell extracts (lanes 2 and 9) suggesting that indeed NTH1 is a major detectable DNA glycosylase activity towards pyrimidine-derived hydantoins in HeLa cells. Similar results were obtained when using the 5′-[^32^P]-labelled DHU•G oligonucleotide duplex as a substrate (Figure 7C).

Fanconi anemia (FA) is a recessive cancer prone syndrome featuring bone marrow failure, hypersensitivity to interstrand DNA cross-links (ICLs) and also to ionizing radiation and oxidative stress [43], [44]. Recently we demonstrated that the immortalized lymphoid cells of FA complementation Group A, C and D2 have decreased level of the NEIL1 protein, whereas, the cellular level of APE1 was similar to that in normal healthy cells [45]. In light of these observations we examined the incision activities of FA complementation Group C (FA-C) cell-free extracts towards 5′-[^32^P]-labelled 5OH-Hyd•G oligonucleotide duplex. As a control for normal non-FA cells we used AHH1 (*WT*) cells and FA-C cells complemented with a plasmid expressing FANCC protein (FA-C+FANCC). FA-C extracts exhibit somewhat decreased DNA glycosylase activity on 5OH-Hyd•G as compared to AHH1 and FA-C+FANCC extracts suggesting that FA-C cells are impaired in the repair of 5OH-Hyd residues (Supporting Information Figure S5). Since NTH1 is a major DNA glycosylase for 5OH-Hyd residues, we measured the NTH1 protein level in FA-C cells. Western blot analysis did not reveal any difference in NTH1 level in extracts from AHH1, FA-C and FA-C+FANCC cells (data not shown). At least three DNA glycosylases can initiate BER for 5OH-Hyd residues in human cells: NTH1, NEIL1 and NEIL2. The cleavage fragments generated by the extracts under BER condition contain 3′-OH group (Figure 7A) and do not migrate as β and β,δ-elimination products generated by pure NTH1 and NEIL1 (lanes 5–6). We may propose that cleavage fragment with 3′-OH group occur due to robust 3′-diesterase and 3′-phosphatase activities in human cell-free extracts. Taken together, these results may suggest deficiency in the repair of oxidative DNA damage in FA cells, however, the observed difference between FA and normal cells was very small (Supporting Information Figure S5).

## Discussion

Oxidized bases are the major endogenous DNA lesions that can accumulate during aging. Purine and pyrimidine moieties of the respective nucleosides undergo oxidative degradation, resulting in a number of modified bases that can be highly mutagenic when present in DNA. 5OH-Hyd and 5OH-5Me-Hyd residues have been shown to be major oxidation decomposition products of cytosine and thymine, respectively [23], [46], [47]. Cells evolved several repair mechanisms to remove oxidized bases from the genome. In the present study, we investigated whether the AP endonucleases involved in the NIR pathway recognize the pyrimidine-derived hydantoins in duplex DNA. The results show for the first time that Nfo, Apn1 and APE1 can incise, in a DNA glycosylase-independent manner, duplex DNA containing both 5OH-Hyd and 5OH-5Me-Hyd residues.

Previous studies of the mechanism of the AP endonuclease-catalyzed nucleotide incision activities were mainly based on the analysis of migration pattern of cleavage fragments in denaturing PAGE [3], [41]. Here, to provide insight into the mechanism of NIR activity we analyzed the AP endonuclease-generated cleavage fragments by MALDI-TOF mass spectrometry. The advantage of MALDI-TOF MS analysis is that it permits simultaneous measurements of every DNA products including non-labelled complementary strand, upstream and downstream cleavage fragments. As expected, the results obtained by MALDI-TOF MS perfectly confirmed those obtained by the denaturing PAGE separation technique: all AP endonucleases tested (*i*) incise the oligonucleotide duplexes 5′ next to 5OH-Hyd and 5OH-5Me-Hyd residues generating 3′ downstream cleavage fragments still containing 5′-terminal damaged nucleotide; (*ii*) degrade 5′ upstream cleavage fragments by their non-specific 3′→5′ exonuclease activity (Figure 2). Importantly, the MALDI-TOF MS analysis shed new light on the mechanism of nucleotide incision activity on the hydantoins by demonstrating that 5OH-Hyd and 5OH-5Me-Hyd residues in DNA undergo degradation into ureido residues during and/or after incubation with the AP endonucleases. MS data revealed that cleavage of 5OH-Hyd•G and 5OH-5Me-Hyd•A oligonucleotide duplex by all AP endonucleases tested generate DNA fragments containing 5′-terminal ureido residues. Indeed, the denaturing PAGE analysis demonstrated that 3′ downstream cleavage fragments, derived from the treatment of 5OH-Hyd•G, migrate faster than 14-mer size marker fragment but still slower than 13-mer size marker and DNA glycosylase-generated fragments suggesting that 5′-terminal hydantoin residue may undergo partial decomposition (Figure 1B). Formation of ureido residues during AP endonuclease treatment does not depend on reaction condition and incubation time. Furthermore, the co-incubation of 5OH-5Me-Hyd•A duplex with Nfo and APE1 did not increase yield of cleavage fragments containing ureido residues (Supporting Information Figure S1). Interestingly, the oxidized pyrimidine bases can undergo ring-chain tautomerism at C6-N1 or C5-N1 bond resulting in formation of acyclic linear structures which could be chemically less stable [48], [49]. Loss of the base stacking stabilization after duplex incision next to damaged base might change the equilibrium of hydantoins ring-chain tautomerism into the less stable open form. However, MS analysis of P1 nuclease digestion of DNA containing both 5OH-Hyd and/or 5OH-5Me-Hyd residues did not reveal any modification of the hydantoin moiety in the nucleosides [30], [36].

Previously, we proposed that the NIR activity requires a more tight binding of the AP endonucleases to DNA substrate containing an oxidatively damaged base, as a consequence APE1 has low turnover rate on αdA-containing DNA substrate as compared to AP site DNA [13], [50]. This tight mode of binding may enable recognition of oxidized bases in duplex DNA by the AP endonucleases by creating specific interactions of active site amino acid residues with a damaged base. Interestingly, when acting upon 5OH-5Me-Hyd•A, APE1 generates cleavage fragment containing only 5′-ureido nucleotides whereas Nfo and Apn1 produce two fragments containing either 5′-5OH-5Me-Hyd or 5′-ureido nucleotides (Figure 1). These results indicate that in contrast to Nfo and Apn1, APE1 cannot incise 5OH-5Me-Hyd•A duplex but rather ureido-containing oligonucleotide duplex. Since, NIR-deficient APE1 mutants cannot cleave the hydantoin-containing duplexes (Supporting Information Figure S1A) and that ureido residue is not present in the non-treated oligonucleotides it is tempting to speculate that under NIR condition APE1 may promote the conversion of 5OH-5Me-Hyd to ureido residue via interactions between its active site amino acids and the damaged pyrimidine. When APE1 binds to DNA it may convert part of 5OH-5Me-Hyd•A to Ureido•A duplex, this would enable APE1 to cut 5′ next to ureido residue generating the observed cleavage fragment with 5′-terminal ureido nucleotide. This is not possible under the BER+Mg^2+^ condition (in the presence of 5 mM MgCl_2_) since under this condition APE1 cannot bind to DNA substrate in the tight manner and catalyze the NIR activity (Supporting Information Figure S2A). Nfo and Apn1 could also promote the conversion of hydantoin to ureido residue by binding to 5OH-5Me-Hyd•A duplex since they also generate ureido residue after reaction. Interestingly, the co-incubation of 5OH-5Me-Hyd•A duplex with Nfo and APE1 did not increase yield of cleavage fragments containing ureido residues (Supporting Information Figure S1). Furthermore, ureido residues can be detected by MS after incubation of the 5OH-Hyd•G duplex with all AP endonucleases tested which may suggest conversion of 5OH-Hyd to ureido residue upon enzyme binding to DNA (Figure 2A,C). Hence, we may speculate that the formation of ureido residues in DNA might be a consequence of both chemical instability of the hydantoins and non-covalent interactions of a damaged base with active site amino acid residues upon AP endonuclease binding. Nevertheless, it should be noted that the degradation of the hydantoins to ureido during or after AP endonuclease-catalyzed cleavage of duplex DNA substrate does not affect removal of the dangling nucleotide residue during reconstitution of the NIR pathway *in vitro*, which leads to the restoration of a damage-free duplex oligonucleotide.

In previous studies, we characterized substrate specificities of the bacterial, yeast and human AP endonucleases towards damaged pyrimidines such as DHU, DHT and 5OHC and demonstrated that *in vitro* the AP endonucleases are more efficient than the DNA glycosylases/AP lyases [13], [14], [51]. In the present work, analysis of kinetic parameters showed that incision of 5OH-Hyd•G by Nfo, Apn1 and APE1 are highly efficient implying that the NIR pathway can efficiently compete with BER in the removal of 5OH-Hyd residues in DNA *in vivo*. In contrast, the kinetics parameters of the cleavage of 5OH-5Me-Hyd•A by eukaryotic AP endonucleases Apn1 and APE1 were inefficient as compared to Nfo and DNA glycosylases suggesting that in eukaryotes the majority of 5OH-5Me-Hyd residues would be removed rather in the BER pathway. Interestingly, among all human DNA repair enzymes tested human NTH1 DNA glycosylase has the highest k_cat_/K_M_ value for incision of 5OH-5Me-Hyd•A substrate. Therefore, excision of 5OH-5Me-Hyd residues by NTH1 would rather initiate short-patch BER pathway similar to excision of 8oxoG residues by hOGG1 [52]. Here, based on a new substrate specificity of APE1 we performed a complete *in vitro* reconstitution of the human NIR pathway for 5OH-Hyd•G duplex oligonucleotides using purified proteins. Incubation of a 5OH-Hyd•G duplex in the presence of APE1, FEN1, POLβ and LIG1 generated a free of 5OH-Hyd residues, full-length oligonucleotide (Figure 4). Interestingly, we did not observed futile repair of 5OH-Hyd•G duplex in the presence of DNA ligase activity suggesting that the repair of APE1-generated single-strand breaks should be accomplished through the removal of 5′-dangling nucleotide in the long-patch NIR pathway. Overall, these results demonstrate that 5OH-Hyd residues can be processed in a DNA glycosylase-independent manner via the NIR pathway.

Data obtained with the purified proteins support the physiological relevance of the AP endonuclease-catalyzed nucleotide incision activity on DNA containing pyrimidine-derived hydantoins. To further investigate the role of various DNA repair pathways, we measured the AP endonuclease and DNA glycosylase activities in cell-free extracts from *E. coli* and human cells. In *E. coli* cell-free extracts we detected mainly DNA glycosylase activities with Nth and Nei being major DNA glycosylases responsible for incision of 5OH-Hyd•G and 5OH-5Me-Hyd•A duplexes and little NIR activity (Figure 5). Although, the Nfo-catalyzed NIR activity towards 5OH-Hyd•G and 5OH-5Me-Hyd•A can be strongly induced by paraquat up to the level similar to those observed for DNA glycosylases (Supporting Information Figure S4). Interestingly, it was shown that *E. coli nth nei* mutants are hypersensitive to the lethal effects of ionizing radiation [53] and hydrogen peroxide [54], implying potential role of pyrimidine-derived hydantoins as lethal oxidative lesions in DNA. In the case of human cell-free extracts, depending on the reaction conditions either NIR+Zn^2+^ and/or BER+EDTA activities were detected (Figure 6A,C). Using small RNA silencing we demonstrated that the alternative DNA glycosylase-independent repair of 5OH-Hyd and 5OH-5Me-Hyd residues in duplex DNA depends upon APE1 thus substantiating the biological role of APE1-catalyzed NIR pathway in human cells (Figure 6). Recently, it has been demonstrated that Nei and NEIL1 mediated excision of 5OH-5Me-Hyd can result in an unproductive DNA–protein covalent (DPC) complex which hides the lesion from repair and represents more complex bulky lesion [55]. This observation further substantiates the biological role of NIR as an alternative pathway which avoids the generation of genotoxic intermediates during repair of the hydantoin DNA lesions.

Under BER (BER+EDTA and BER+Mg^2+^) conditions, three human DNA glycosylases can excise 5OH-Hyd and 5OH-5Me-Hyd residues hence contributing to the redundancy in DNA repair pathways that may back-up each other and/or act preferably depending on chromatin context, DNA damage signalling pathway and various cellular regulation mechanisms. Study of the BER activities in HeLa cell extracts demonstrated that NTH1 is a major detectable DNA glycosylase activity towards 5OH-Hyd and 5OH-5Me-Hyd residues in DNA (Figure 7). Surprisingly, we were not able to detect NEIL1 and NEIL2 activities using our hydantoin-DNA substrates possibly due to a strong 3′-repair diesterase activity present in human cell-free extracts. Human FA cells appear to be a highly valuable model to study cellular response to endogenous oxidative DNA damage. Ambient oxygen induces chromosomal instability in FA cells suggesting impaired cellular defence against oxidative DNA damage, furthermore we have recently shown that FA cells have reduced amounts of NEIL1 [45]. Interestingly, here we demonstrated that FA cell-free extracts have slightly reduced BER incision activity towards 5OH-Hyd•G duplex oligonucleotide implying that NEIL1 may serve as a back-up DNA glycosylase to repair pyrimidine-derived hydantoins (Supporting Information Figure S5). Human NTH1 protein has been shown to be able to initiate BER in nucleosome protected DNA [56], while NEIL1 and NEIL2 proteins excise oxidative base lesions in single-stranded and bubble DNA structures, suggesting their functions are coupled to DNA replication and/or transcription processes [57], [58], [59]. 5OH-Hyd and 5OH-5Me-Hyd residues are major oxidative pyrimidine lesions that accumulate in ancient DNA and may also accumulate during long chronic exposure to oxidizing agents [29]. Therefore, it is tempting to speculate that pyrimidine-derived hydantoins in non-transcribed heterochromatin DNA regions are main targets to NTH1 and APE1 but not to NEIL1, suggesting biological function of the NTH1-catalyzed BER and the APE1-NIR in the global genome repair pathway for pyrimidine-derived hydantoins elsewhere in genome.

## Materials and Methods

### Oligonucleotides, proteins and antibodies

Sequences of the oligonucleotides used in the present work are shown in Table 2. The 22-mer oligonucleotides containing 5OH-Hyd and 5OH-5Me-Hyd were synthesized as previously described [30], [36]. The 19-mer oligonucleotide containing 5,6-dihydroxy-5,6-dihydrothymidine (or thymine glycol) (Tg) was kindly provided by Hiroshi Ide (Hiroshima University, Japan) [60]. All other oligonucleotides were purchased from Eurogentec (Seraing, Belgium). Prior to enzymatic assays oligonucleotides were either 5′-end labelled by T4 polynucleotide kinase (New England Biolabs) in the presence of [γ-^32^P]-ATP (3,000 Ci/mmol-1) (PerkinElmer), or 3′-end labelled by terminal deoxynucleotidyl transferase (New England Biolabs) in the presence of [α-^32^P]-3′-dATP (Cordycepin 5′-triphosphate, 5,000 Ci/mmol^−1^) (PerkinElmer) as recommended by the manufacturers. Radioactively labelled oligonucleotides were desalted with a Sephadex G-25 column equilibrated in water and then annealed with corresponding complementary strands for 3 min at 65°C in a buffer containing 20 mM HEPES-KOH (pH 7.6), 50 mM KCl. The resulting duplex oligonucleotides are referred to as X•G (C,A,T), respectively, where X is a modified residue.

**Table 2:** Sequence of the oligonucleotides used in the study, where X is a corresponding modified residue: 5OH-Hyd, 5OH-5Me-Hyd, DHU or Tg.

Name	Sequence 5′→3′	Ref.
X-DG	d(CACTTCGGAXTGTGACTGATCC)	[34]
DG-Y	d(GGATCAGTCACAATCCGAAGTG)	[34]
X-RT	d(TGACTGCATAXGCATGTAGACGATGTGCAT)	[3]
RT-Y	d(ATGCACATCGTCTACATGCNTATGCAGTCA)	[3]
Tg 34-mer	d(AAATACATCGTCACCTGGGXCATGTTGCAGATCC)	This work
19Tg-IW 19-mer	d(ACAGACGCCAXCAACCAGG)	[60]
DG-13C-5P	p-d(CTGTGACTGATCC)	This work
DG-13T-5P	p-d(TTGTGACTGATCC)	This work
DG-12-5P	p-d(TGTGACTGATCC)	This work
si-APE1(sense)	UACUCCAGUCGUACCAGACCU	[61]
si-APE1 (antisense)	GUCUGGUACGACUGGAGUACC	[61]
si-NTH1(sense)	GGAGCAAGGUGAAAUACAUCAAGCA	[42]
si-NTH1 (antisense)	UGCUUGAUGUAUUUCACCUUGCUCC	[42]
si-Control (sense)	ACUAUGUAUAGGAGUACGCTT	[14]
si-Control (antisense)	GCGUACUCCUAUACAUAGUTT	[14]

N is an opposite regular base in the complementary strand.

The siRNAs sequences used to decrease APE1 and NTH1 in HeLa cells have been taken from previously described studies [42], [61]. The siRNA specific to mouse major AP endonuclease, APEX, was used as negative control in both cases. Collection of the purified DNA glycosylases and AP endonucleases and human FEN1 was from the laboratory stock. The purified human POLβ and LIG1 proteins were purchased from Trevigen (Gaithersburg, USA) and Enzymax (Lexington, USA), respectively. Polyclonal rabbit antibodies against human APE1 and NTH1 proteins were from Eurogentec and Alpha Diagnostic (Interchim, Montlucon, France), respectively.

### DNA repair assays

The standard reaction mixture (20 µL) contained 5 nM of ^32^P-labelled 5OH-Hyd•G and 5OH-5Me-Hyd•A oligonucleotide duplexes. Assays for the DNA glycosylases activities were performed in a “BER+EDTA” buffer containing 50 mM KCl, 20 mM HEPES-KOH (pH 7.6), 0.1 mg·mL^−1^ BSA, 1 mM DTT and 1 mM EDTA and 2.5 nM of a pure protein, unless otherwise stated. The release of 5OH-Hyd and 5OH-5Me-Hyd base adducts was measured by the cleavage of the oligonucleotide containing a single lesion at a defined position. For the monofunctional DNA glycosylases, the abasic sites left after damaged base excision action were cleaved by light piperidine treatment (10% (v/v) piperidine at 37°C for 10 min).

To measure kinetic parameters, range of duplex oligonucleotide substrate concentrations varied from 0.1 to 10 times the K_M_ (generally from 2.5 nM to 250 nM) were incubated under respective standard reaction conditions with limiting amounts of enzymes (0.25 nM Nfo, 0.5 nM Apn1, 0.5 nM APE1, 2.5 nM NEIL1 and 20 nM NTH1) for 5–10 min at 37°C, unless otherwise stated. For *K*_M_ and *k*_cat_ determination, the linear velocities were plotted against substrate concentration and the hyperbolic curve obtained fit to a rectangular hyperbola by least-squares non-linear regression method. Apparent values were obtained for the Michaelis constant, *K*_M_, and the *V*_max_ for cleavage; *k*_cat_ was calculated by dividing the *V*_max_ by the enzyme concentration. At least three independent experiments were performed for all analyses.

The standard reaction mixture (20 µL) for the NIR activity contained 50 mM KCl, 20 mM HEPES-KOH (pH 7.6), 0.1 mg•mL^−1^ BSA and 1 mM DTT for the Nfo protein or the same buffer supplemented with 5 mM MgCl_2_ for the Apn1 protein. The activity of APE1 protein was tested either in the “BER+Mg^2+^” buffer containing 100 mM KCl, 20 mM HEPES-KOH (pH 7.6), 0.1 mg•mL^−1^ BSA, 1 mM DTT and 5 mM MgCl_2_ which is optimal for its AP endonuclease activity or in the “NIR buffer” containing 50 mM KCl, 20 mM HEPES-KOH (pH 6.9), 0.1 mg•mL^−1^ BSA, 1 mM DTT and 0.1 mM MgCl_2_ which is optimal for its nucleotide incision activity. When measuring APE1-NIR activity in the cell-free extracts MgCl_2_ was replaced with ZnCl_2_ [14].

The standard incision assay in cell-free extracts (20 µl) was performed with 5 nM ^32^P-labelled DNA substrate either in the standard DNA glycosylase BER+EDTA reaction buffer: 50 mM KCl, 20 mM HEPES-KOH (pH 7.6), 0.1 mg•mL^−1^ BSA, 1 mM DTT and 1 mM EDTA or in the zinc-containing “NIR+Zn^2+^ buffer” optimal for the APE1-catalyzed NIR activity in extracts: 50 mM KCl, 20 mM HEPES-KOH (pH 6.8), 0.1 mg•mL^−1^ BSA and 1 mM DTT and 0.1 mM ZnCl_2_ [14]. Reaction mixtures were supplemented with either 0.5 µg of human cell-free extract or 3 µg of *E. coli* cell extracts and incubated for 60 min at 37°C, unless otherwise stated.

The reactions were stopped by adding 10 µL of a solution containing 0.5% SDS and 20 mM EDTA and then desalted by hand-made spin-down columns filled with Sephadex G25 (Amersham Biosciences) equilibrated in 7.5 M urea. Purified reaction products were heated at 65°C for 3 min and separated by electrophoresis in denaturing 20% (w/v) polyacrylamide gels (7 M urea, 0.5× TBE, 42°C). Gels were exposed to a Fuji FLA-3000 Phosphor Screen and analyzed using Image Gauge V3.12 software.

*In vitro* reconstitution of the human NIR pathway for 5OH-Hyd residues was carried out as described previously [41]. Briefly, 5 nM of 3′-[^32^P]-labelled 5OH-Hyd•G duplex was incubated in the presence of 5 nM APE1, 0.1 units POLβ, 2 nM FEN1 and 5 nM LIG1 in buffer (20 µL) containing 50 mM HEPES-KOH (pH 7.2), 30 mM NaCl, 0.1 mg/mL BSA, 2 mM DTT, 50 µM dNTPs, 2 mM ATP and 3 mM MgCl_2_ for 60 min at 25°C. To reveal non-repaired 5OH-Hyd residues, left after incubation with human proteins, additional treatment with *E. coli* Nei DNA glycosylase was performed. For this purpose, an aliquot from reaction mixture was incubated in 20 µL of DNA glycosylase buffer with 5 nM Nei for 10 min at 37°C. Reaction products were analyzed as described above.

### MALDI-TOF mass spectrometry analyses of the NIR pathway

Typically, 40 pmol of lesion containing oligonucleotide duplexes (in 20 µL) were incubated with the AP endonucleases (100 ng of APE1 and/or Nfo) in the appropriate “NIR reaction buffer” (see above) at 37°C for 1 h. The products were desalted on a MicroSpin G-25 column (GE Healthcare), prior subjection to the MALDI-TOF MS measurements. The latter MALDI mass spectra in the negative mode were obtained on a time-of-flight Biflex mass spectrometer (Bruker, Wissembourg, France) equipped with a 337 nm nitrogen laser and pulsed delay source extraction. The matrix was prepared by dissolving 3-hydroxypicolinic acid in 10 mM ammonium citrate buffer and a small amount of Dowex-50 W 50×8–200 cation exchange resin (Sigma). Sample (1 µL) was added to matrix (1 µL) and the resulting solution was made homogeneous by stirring. The resulting sample was placed on the target plate and allowed to dry. Spectra were calibrated using reference oligonucleotides of known masses.

### *E. coli* strains

*E. coli* AB1157 (*leuB6 thr*-*1Δ(gpt-proA2) hisG4 argE3 lacY1 galK2 ara-14 mtl-1 xyl-5 thi-1 tsx-33 rpsL31 supE44 rac*) (*WT*) and its isogenic derivatives MS2000 (*nei::spc^R^ (fpg::kn^R^, X::tet^R^*)) (*where X::tet^R^ is the insertion of transposon Tn10 with an unknown location used as a co-transducible marker with the fpg::Kn^R^*), BH130 (*nfo::kn^R^*), BH150 (*nth::kn^R^*), BH160 (*nth::kn^R^ fpg::kn^R^*) were from laboratory stock and SW2-8 (*Δnei::Cm^R^*) and SW2-38 (*nth::kn^R^ Δnei::cm^R^*) isogenic to BW35 (KL16) were gifts from S. Wallace (University of Vermont, U.S.A).

Cell-free extracts were prepared as previously described [3]. Briefly, *E. coli* cultures were grown to an OD_600_ of 1.0 in the presence or absence of 0.25 mg/mL paraquat (+P) to induce Nfo [62]. Following cells were harvested by centrifugation, washed and lysed with 10 mg/mL lysozyme in a buffer containing 0.1 M HEPES-KOH (pH 7.5), 400 mM KCl, 0.1 mM EDTA. Cell lysates were clarified by centrifugation at 14000 rpm for 15 min at 4°C and then aliquoted and stored at -80°C until use.

### Cell culture and silencing of APE1 and NTH1 expression

HeLa cells (ATCC #CCL-2, U.S.A.) were routinely grown at 37°C in 5% CO_2_ in Dulbecco minimal essential medium supplemented with 10% foetal calf serum, 2 mM glutamine, 100 U/mL penicillin and 100 mg/mL streptomycin. The EBV-transformed lymphoblastoid cell lines from wild-type (AHH1), FA-C (HSC536), FA-C expressing neomycin (HSC536N), FA-C expressing neomycin and FANCC (HSC536N/FANCC), lymphoblast cells (kindly provided by Dr F. Rosselli, Institut Gustave Roussy, Villejuif, France) were routinely grown at 37°C in 5% CO_2_ in RPMI medium supplemented with 12% foetal calf serum, 2 mM glutamine, 100 U/mL penicillin and 100 mg/mL streptomycin as described [63]. HSC-536N and HSC536N/FANCC cell lines are retroviral-transduced lymphoblasts and were previously described [64].

HeLa cells were transfected with the siRNA oligonucleotides using Lipofectamine 2000 (Invitrogen, France) according to the manufacturer's instructions. Cells were plated at 2×10^6^ cells per Petri dish, incubated for 18 h and then transfected with the specific siRNA. Transfection efficiency was monitored by co-transfection of control cells with pmaxGFP vector (Amaxa, Germany) (data not shown). After 72 h, cells were collected and whole cell-free extracts were prepared as described [3]. Briefly, the cells pellet was washed in ice-cold PBS and incubated for 15 min at 4°C with rocking in the lysis buffer containing 1 M KCl, 80 mM HEPES (pH 7.6), 0.1 mM EDTA, 2 mM DTT, 0.3% NP-40 and protease inhibitor cocktail (Complete EDTA-free, Roche). After centrifugation at 65000 rpm for 1 h at 4°C, the supernatants were collected and stored in 50% glycerol at −20°C for immediate use or at −80°C for longer storage. To measure the protein level the antibodies' revealed bands in western blots were quantified by densitometry with ImageJ software (National Institutes of Health, Bethesda, MD, USA). Relative repair activities in the cell-free extracts were normalized to the level of α-actin in western blots.

## Supporting Information

Figure S1 **The AP endonuclease activities towards 5OH-5Me-Hyd•A oligonucleotide duplex.** The 3′-[^32^P]-labelled 5OH-5Me-Hyd•A oligonucleotide duplex was incubated with purified WT and mutant Nfo and APE1 under either standard condition “pH 7.6” for Nfo, or “NIR+Mg^2+^” same as “pH 6.9, 0.1 mM MgCl_2_” for APE1, or standard condition “BER+EDTA” same as “pH 7.6, 1 mM EDTA” for Fpg. (**A**) Denaturing PAGE analysis of the cleavage products by WT and mutant AP endonucleases. Nfo-G149G is a highly NIR-deficient mutant of Nfo; R177A and K98E are APE1 mutants with a slightly reduced NIR activity; D308A is APE1 mutant with highly reduced NIR activity; K98A/R185A is APE1 double mutant with highly reduced NIR activity; ΔN61 is a truncated APE1 mutant lacking the N-terminal 61 residues with a slightly reduced NIR activity; D210N is a catalytically inactive APE1 mutant. “5′-p-MeHyd” denotes 14-mer cleavage fragment containing 5′-terminal 5OH-5Me-Hyd residue; “5′-p-Z” denotes 14-mer cleavage fragment containing 5′-terminal ureido residue. (**B**) Cleavage of 5OH-5Me-Hyd•A oligonucleotide duplex by Nfo, APE1 and Fpg after 10 and 45 min incubation. “P1” and “5MeHyd-13mer” denotes 14-mer cleavage fragment containing 5′-terminal 5OH-5Me-Hyd residue; “P2” and Y-13mer” denotes 14-mer cleavage fragment containing 5′-terminal ureido residue; “P3” and “13mer” denotes 13-mer cleavage fragment containing 5′-phosphate residue. For details see Materials and Methods.
(JPG)

Figure S2 **Comparison of the AP endonuclease activities towards urea, hydantoins and thymine glycol containing substrates.** 5′-[^32^P]-labelled oligonucleotide duplexes were incubated with the purified WT and mutant Nfo and APE1 AP endonucleases under standard BER and NIR conditions. Nfo-G149G is a highly NIR-deficient mutant of Nfo; R177A and K98E are APE1 mutants with a slightly reduced NIR activity; D308A is APE1 mutant with highly reduced NIR activity; K98A/R185A is APE1 double mutant with highly reduced NIR activity; ΔN61 is a truncated APE1 mutant lacking the N-terminal 61 residues with a slightly reduced NIR activity; D210N is a catalytically inactive APE1 mutant. (**A**) Denaturing PAGE analysis of the cleavage products of Urea-RT•T, 5OH-Hyd•G and 5OH-5Me-Hyd•A oligonucleotide duplexes. Lane 1, control non-treated Urea-RT•T; lane 2, as 1 but with Nfo; lane 3, as 1 but with APE1 under NIR condition; lane 4, as 1 but with APE1 under BER condition; lane 5, as 1 but with Xth; lanes 6–10, same as 1–5 but with 5OH-Hyd•G as a substrate; lanes 11–15, same as 1–5 but with 5OH-5Me-Hyd•A as a substrate. (**B**) Denaturing PAGE analysis of the APE1-cleavage products of 5′-[^32^P]-labelled 30-mer, 34-mer and 19-mer Tg•A, oligonucleotide duplexes under NIR condition. Following reaction buffers were used for the AP endonucleases: buffer for Nfo contained 20 mM HEPES-KOH, pH 7.6, 50 mM KCl, 0.1 mg/mL BSA and 1 mM DTT treatment; buffer for APE1-NIR contained 20 mM HEPES-KOH, pH 6.9, 50 mM KCl, 0.1 mg/mL BSA, 1 mM DTT and 0.1 mM MgCl_2_; buffer for APE1-BER contained 20 mM HEPES-KOH, pH 7.6, 100 mM KCl, 0.1 mg/mL BSA, 1 mM DTT and 5 mM MgCl_2_. Reaction buffer for Xth contained 20 mM HEPES-KOH, pH 7.6, 100 mM KCl, 0.1 mg/mL BSA, 1 mM DTT and 5 mM CaCl_2_. Lane 1, control non-treated 30-mer Tg•A; lane 2, as 1 but with WT APE1; lane 3, as 1 but with APE1-K98E mutant; lane 4, control non-treated 34-mer Tg•A; lane 5, as 4 but with WT APE1; lane 6, as 4 but with APE1-K98E mutant; lane 7, control non-treated 19-mer 19Tg-IW•A; lane 8, as 7 but with WT APE1; lane 9, as 7 but with APE1-K98E mutant. (**C**) Denaturing PAGE analysis of the WT and mutant Nfo and APE1 activities on 5′-[^32^P]-labelled RT-THF•T and Urea-RT•T oligonucleotide duplexes. Lane 1, control non-treated RT-THF•T; lane 2, as 1 but with WT Nfo; lane 3, as 1 but with NIR-deficient mutant Nfo-G149G; lane 4, as 1 but APE1 under NIR condition; lanes 5–9, as 4 but with APE1-mutants; lanes 10–18, same as lanes 1–9 but with RT-Urea•T duplex as a substrate. For details see Materials and Methods.
(JPG)

Figure S3 **Comparison of the AP endonucleases and DNA glycosylases activities towards pyrimidine-derived hydantoins.** Enzyme concentration dependence activity curves on 5′-[^32^P]-labelled oligonucleotide duplexes containing hydantoins (**A**) Nfo, Apn1 and APE1 acting on 5OH-Hyd•G; (**B**) Nfo, Apn1 and APE1 acting on 5OH-5Me-Hyd•A; (**C**) NTH1 and NEIL1 acting on 5OH-Hyd•G; (**D**) NTH1 and NEIL1 acting on 5OH-5Me-Hyd•A. For details see Materials and Methods.
(JPG)

Figure S4 **DNA repair activities towards 5OH-Hyd•G duplex in paraquat-induced** ***E. coli*** **cell-free extracts.** 3′-[α-^32^P]-ddATP-labelled oligonucleotide duplexes were incubated with either 3 µg of cell-free extract or limited amount of a purified protein in the standard DNA glycosylase reaction “BER+EDTA” buffer for 30 min at 37°C. (**A**) Denaturing PAGE analysis of the reaction products of 5OH-Hyd•G oligonucleotide. Lanes 1 and 12, control 5OH-Hyd•G with no enzyme; lanes 2–3 and 13–14, 5OH-Hyd•G incubated with the purified proteins; lanes 4–11, 5OH-Hyd•G incubated with control non-induced extracts; lanes 15–22, 5OH-Hyd•G incubated with paraquat-induced extracts. (**B**) Graphic representation of the mean values of DNA repair activities on 5OH-Hyd•G. (**C**) Denaturing PAGE analysis of the reaction products of 5OH-5Me-Hyd•G oligonucleotide. Lanes 1 and 12, control 5OH-5Me-Hyd•A with no enzyme; lanes 2–3 and 13–14, 5OH-5Me-Hyd•A incubated with the purified proteins; lanes 4–11, 5OH-5Me-Hyd•A incubated with control non-induced extracts; lanes 15–22, 5OH-5Me-Hyd•A incubated with paraquat-induced extracts. (**D**) Graphic representation of the mean values of DNA repair activities on 5OH-5Me-Hyd•A. DNA glycosylase (BER) and AP endonuclease-catalyzed (NIR) incisions were calculated by measuring amount of 13-mer and 14-mer products, respectively. The background values representing control oligonucleotides degradation in absence of enzyme in lanes 1 and 12 were subtracted. “*E. coli extracts+P*” indicates that the expression of Nfo was induced by exposure of cell culture to 0.25 mg/mL of paraquat. For details see Materials and Methods.
(JPG)

Figure S5 **DNA repair activities towards 5-OH-Hyd residues in the extracts of Fanconi complementation group C cells.** 5′-[^32^P]-labelled 5OH-Hyd•G oligonucleotide duplex was incubated with 20 µg of the extracts prepared from AHH1, FA-C and FA-C+FANCC cells. (**A**) Denaturing PAGE analysis of the reaction products. Lane 1, 5OH-Hyd•G treated with NEIL1; lane 2, 5OH-Hyd•G treated with NTH1; lane 3, control 5OH-Hyd•G no treatment; lanes 4–6, 5OH-Hyd•G treated with AHH1 extracts; lanes 7–9, 5OH-Hyd•G treated with FA-C extracts; lanes 10–12, 5OH-Hyd•G treated with FA-C+FANCC extracts. The arrows denote the position of the 22-mer, 9-mer fragment with 3′-dRP residue (9-dRP) and 9-mer fragment with 3′-phosphate residue (9-P). (**B**) Graphic representation of the mean values of DNA repair activities in extracts. For details see Materials and Methods.
(JPG)

## References

[1] Cadet J, Douki T, Gasparutto D, Ravanat JL (2003). Oxidative damage to DNA: formation, measurement and biochemical features.. Mutat Res.

[2] Sancar A, Lindsey-Boltz LA, Unsal-Kacmaz K, Linn S (2004). Molecular mechanisms of mammalian DNA repair and the DNA damage checkpoints.. Annual Review of Biochemistry.

[3] Ischenko AA, Saparbaev MK (2002). Alternative nucleotide incision repair pathway for oxidative DNA damage.. Nature.

[4] Redrejo-Rodríguez M, Ishchenko AA, Saparbaev MK, Salas ML, Salas J (2009). African swine fever virus AP endonuclease is a redox-sensitive enzyme that repairs alkylating and oxidative damage to DNA.. Virology.

[5] Bandaru V, Zhao X, Newton MR, Burrows CJ, Wallace SS (2007). Human endonuclease VIII-like (NEIL) proteins in the giant DNA Mimivirus.. DNA Repair (Amst).

[6] Georg J, Schomacher L, Chong JP, Majernik AI, Raabe M (2006). The Methanothermobacter thermautotrophicus ExoIII homologue Mth212 is a DNA uridine endonuclease.. Nucleic Acids Res.

[7] Hitomi K, Iwai S, Tainer JA (2007). The intricate structural chemistry of base excision repair machinery: implications for DNA damage recognition, removal, and repair.. DNA Repair (Amst).

[8] Breslin C, Caldecott KW (2009). DNA 3′-phosphatase activity is critical for rapid global rates of single-strand break repair following oxidative stress.. Mol Cell Biol.

[9] Fromme JC, Banerjee A, Verdine GL (2004). DNA glycosylase recognition and catalysis.. Curr Opin Struct Biol.

[10] Zharkov DO (2008). Base excision DNA repair.. Cell Mol Life Sci.

[11] Ide H, Tedzuka K, Shimzu H, Kimura Y, Purmal AA (1994). Alpha-deoxyadenosine, a major anoxic radiolysis product of adenine in DNA, is a substrate for *Escherichia coli* endonuclease IV.. Biochemistry.

[12] Ishchenko AA, Ide H, Ramotar D, Nevinsky G, Saparbaev M (2004). Alpha-anomeric deoxynucleotides, anoxic products of ionizing radiation, are substrates for the endonuclease IV-type AP endonucleases.. Biochemistry.

[13] Gros L, Ishchenko AA, Ide H, Elder RH, Saparbaev MK (2004). The major human AP endonuclease (Ape1) is involved in the nucleotide incision repair pathway.. Nucleic Acids Res.

[14] Daviet S, Couve-Privat S, Gros L, Shinozuka K, Ide H (2007). Major oxidative products of cytosine are substrates for the nucleotide incision repair pathway.. DNA Repair (Amst).

[15] Demple B, Harrison L (1994). Repair of oxidative damage to DNA: enzymology and biology.. Annu Rev Biochem.

[16] Xanthoudakis S, Miao G, Wang F, Pan YC, Curran T (1992). Redox activation of Fos-Jun DNA binding activity is mediated by a DNA repair enzyme.. Embo J.

[17] Ishchenko AA, Deprez E, Maksimenko A, Brochon JC, Tauc P (2006). Uncoupling of the base excision and nucleotide incision repair pathways reveals their respective biological roles.. Proc Natl Acad Sci U S A.

[18] Beernink PT, Segelke BW, Hadi MZ, Erzberger JP, Wilson DM (2001). Two divalent metal ions in the active site of a new crystal form of human apurinic/apyrimidinic endonuclease, Ape1: implications for the catalytic mechanism.. J Mol Biol.

[19] Gorman MA, Morera S, Rothwell DG, de La Fortelle E, Mol CD (1997). The crystal structure of the human DNA repair endonuclease HAP1 suggests the recognition of extra-helical deoxyribose at DNA abasic sites.. Embo J.

[20] Vallee BL, Falchuk KH (1993). The biochemical basis of zinc physiology.. Physiol Rev.

[21] Dizdaroglu M, Bauche C, Rodriguez H, Laval J (2000). Novel substrates of Escherichia coli nth protein and its kinetics for excision of modified bases from DNA damaged by free radicals.. Biochemistry.

[22] Breimer LH, Lindahl T (1985). Enzymatic excision of DNA bases damaged by exposure to ionizing radiation or oxidizing agents.. Mutat Res.

[23] Wagner JR, Cadet J (2010). Oxidation reactions of cytosine DNA components by hydroxyl radical and one-electron oxidants in aerated aqueous solutions.. Acc Chem Res.

[24] Riviere J, Bergeron F, Tremblay S, Gasparutto D, Cadet J (2004). Oxidation of 5-hydroxy-2′-deoxyuridine into isodialuric acid, dialuric acid, and hydantoin products.. J Am Chem Soc.

[25] Riviere J, Klarskov K, Wagner JR (2005). Oxidation of 5-hydroxypyrimidine nucleosides to 5-hydroxyhydantoin and its alpha-hydroxy-ketone isomer.. Chem Res Toxicol.

[26] Senturker S, Karahalil B, Inal M, Yilmaz H, Muslumanoglu H (1997). Oxidative DNA base damage and antioxidant enzyme levels in childhood acute lymphoblastic leukemia.. FEBS Letters.

[27] Olinski R, Zastawny T, Budzbon J, Skokowski J, Zegarski W (1992). DNA base modifications in chromatin of human cancerous tissues.. FEBS Letters.

[28] Olinski R, Zastawny TH, Foksinski M, Windorbska W, Jaruga P (1996). DNA base damage in lymphocytes of cancer patients undergoing radiation therapy.. Cancer Letters.

[29] Hoss M, Jaruga P, Zastawny TH, Dizdaroglu M, Paabo S (1996). DNA damage and DNA sequence retrieval from ancient tissues.. Nucleic Acids Research.

[30] Gasparutto D, Ait-Abbas M, Jaquinod M, Boiteux S, Cadet J (2000). Repair and coding properties of 5-hydroxy-5-methylhydantoin nucleosides inserted into DNA oligomers.. Chemical Research in Toxicology.

[31] d'Abbadie M, Hofreiter M, Vaisman A, Loakes D, Gasparutto D (2007). Molecular breeding of polymerases for amplification of ancient DNA.. Nat Biotechnol.

[32] McDonald JP, Hall A, Gasparutto D, Cadet J, Ballantyne J (2006). Novel thermostable Y-family polymerases: applications for the PCR amplification of damaged or ancient DNAs.. Nucleic Acids Research.

[33] Briggs AW, Stenzel U, Meyer M, Krause J, Kircher M (2010). Removal of deaminated cytosines and detection of in vivo methylation in ancient DNA.. Nucleic Acids Research.

[34] Gasparutto D, Muller E, Boiteux S, Cadet J (2009). Excision of the oxidatively formed 5-hydroxyhydantoin and 5-hydroxy-5-methylhydantoin pyrimidine lesions by Escherichia coli and Saccharomyces cerevisiae DNA N-glycosylases.. Biochimica et Biophysica Acta.

[35] Breimer LH, Lindahl T (1984). DNA glycosylase activities for thymine residues damaged by ring saturation, fragmentation, or ring contraction are functions of endonuclease III in Escherichia coli.. J Biol Chem.

[36] Muller E, Gasparutto D, Lebrun C, Cadet J (2001). Site-Specific Insertion of the (5R*) and (5S*) Diastereoisomers of 1-[2-Deoxy-β-D-*erythro*-pentofuranosyl]-5-hydroxyhydantoin into Oligodeoxyribonucleotides.. European Journal of Organic Chemistry.

[37] Dubey I, Pratviel G, Robert A, Meunier B (2001). Convenient method for the preparation of 2′-deoxyribosylurea by thymidine oxidation and NMR study of both anomers.. Nucleosides Nucleotides Nucleic Acids.

[38] Toga T, Yamamoto J, Iwai S (2009). Efficient conversion of thymine glycol into the formamide lesion in oligonucleotides.. Tetrahedron Letters.

[39] Guy A, Ahmad S, Téoule R (1990). Insertion of the fragile 2′-deoxyribosylurea residue into oligodeoxynucleotides.. Tetrahedron Letters.

[40] Kow YW, Wallace SS (1985). Exonuclease III recognizes urea residues in oxidized DNA.. Proceedings of the National Academy of Sciences of the United States of America.

[41] Gelin A, Redrejo-Rodriguez M, Laval J, Fedorova OS, Saparbaev M (2010). Genetic and Biochemical Characterization of Human AP Endonuclease 1 Mutants Deficient in Nucleotide Incision Repair Activity.. PLoS One.

[42] Suzuki T, Harashima H, Kamiya H (2010). Effects of base excision repair proteins on mutagenesis by 8-oxo-7,8-dihydroguanine (8-hydroxyguanine) paired with cytosine and adenine.. DNA Repair (Amst).

[43] Moldovan GL, D'Andrea AD (2009). How the fanconi anemia pathway guards the genome.. Annu Rev Genet.

[44] Wang W (2007). Emergence of a DNA-damage response network consisting of Fanconi anaemia and BRCA proteins.. Nat Rev Genet.

[45] Mace-Aime G, Couve S, Khassenov B, Rosselli F, Saparbaev MK (2010). The Fanconi anemia pathway promotes DNA glycosylase-dependent excision of interstrand DNA crosslinks.. Environ Mol Mutagen.

[46] Teoule R, Bert C, Bonicel A (1977). Thymine fragment damage retained in the DNA polynucleotide chain after gamma irradiation in aerated solutions. II.. Radiat Res.

[47] Wagner JR, Decarroz C, Berger M, Cadet J (1999). Hydroxyl-Radical-Induced Decomposition of 2′-Deoxycytidine in Aerated Aqueous Solutions.. Journal of the American Chemical Society.

[48] D'Ham C, Ravanat JL, Cadet J (1998). Gas chromatography-mass spectrometry with high-performance liquid chromatography prepurification for monitoring the endonuclease III-mediated excision of 5-hydroxy-5,6-dihydrothymine and 5,6-dihydrothymine from gamma-irradiated DNA.. J Chromatogr B Biomed Sci Appl.

[49] Jurado J, Saparbaev M, Matray TJ, Greenberg MM, Laval J (1998). The ring fragmentation product of thymidine C5-hydrate when present in DNA is repaired by the Escherichia coli Fpg and Nth proteins.. Biochemistry.

[50] Golan G, Ishchenko AA, Khassenov B, Shoham G, Saparbaev MK (2010). Coupling of the nucleotide incision and 3′→5′ exonuclease activities in Escherichia coli endonuclease IV: Structural and genetic evidences.. Mutat Res.

[51] Ishchenko AA, Sanz G, Privezentzev CV, Maksimenko AV, Saparbaev M (2003). Characterisation of new substrate specificities of *Escherichia coli* and *Saccharomyces cerevisiae* AP endonucleases.. Nucleic Acids Res.

[52] Fortini P, Parlanti E, Sidorkina OM, Laval J, Dogliotti E (1999). The type of DNA glycosylase determines the base excision repair pathway in mammalian cells.. J Biol Chem.

[53] Jiang D, Hatahet Z, Blaisdell JO, Melamede RJ, Wallace SS (1997). *Escherichia coli* endonuclease VIII: cloning, sequencing, and overexpression of the *nei* structural gene and characterization of *nei* and *nei nth* mutants.. J Bacteriol.

[54] Saito Y, Uraki F, Nakajima S, Asaeda A, Ono K (1997). Characterization of endonuclease III (*nth*) and endonuclease VIII (*nei*) mutants of *Escherichia coli* K-12.. J Bacteriol.

[55] Le Bihan YV, Angeles Izquierdo M, Coste F, Aller P, Culard F (2011). 5-Hydroxy-5-methylhydantoin DNA lesion, a molecular trap for DNA glycosylases.. Nucleic Acids Res.

[56] Prasad A, Wallace SS, Pederson DS (2007). Initiation of base excision repair of oxidative lesions in nucleosomes by the human, bifunctional DNA glycosylase NTH1.. Molecular and Cellular Biology.

[57] Dou H, Mitra S, Hazra TK (2003). Repair of oxidized bases in DNA bubble structures by human DNA glycosylases NEIL1 and NEIL2.. Journal of Biological Chemistry.

[58] Zhao X, Krishnamurthy N, Burrows CJ, David SS (2010). Mutation versus repair: NEIL1 removal of hydantoin lesions in single-stranded, bulge, bubble, and duplex DNA contexts.. Biochemistry.

[59] Banerjee D, Mandal SM, Das A, Hegde ML, Das S (2011). Preferential repair of oxidized base damage in the transcribed genes of mammalian cells.. J Biol Chem.

[60] Katafuchi A, Nakano T, Masaoka A, Terato H, Iwai S (2004). Differential specificity of human and *Escherichia coli* endonuclease III and VIII homologues for oxidative base lesions.. J Biol Chem.

[61] Wang D, Luo M, Kelley MR (2004). Human apurinic endonuclease 1 (APE1) expression and prognostic significance in osteosarcoma: enhanced sensitivity of osteosarcoma to DNA damaging agents using silencing RNA APE1 expression inhibition.. Molecular Cancer Therapeutics.

[62] Chan E, Weiss B (1987). Endonuclease IV of Escherichia coli is induced by paraquat.. Proc Natl Acad Sci USA.

[63] Zanier R, Briot D, Dugas du Villard JA, Sarasin A, Rosselli F (2004). Fanconi anemia C gene product regulates expression of genes involved in differentiation and inflammation.. Oncogene.

[64] Walsh CE, Grompe M, Vanin E, Buchwald M, Young NS (1994). A functionally active retrovirus vector for gene therapy in Fanconi anemia group C.. Blood.

