# Supplementary figures and images for: New Insights in the Removal of the Hydantoins, Oxidation Product of Pyrimidines, via the Base Excision and Nucleotide Incision Repair Pathways
Source: PLoS One. 2011 Jul 25;6(7):e21039. doi: 10.1371/journal.pone.0021039 (PMC3143120; doi:10.1371/journal.pone.0021039)

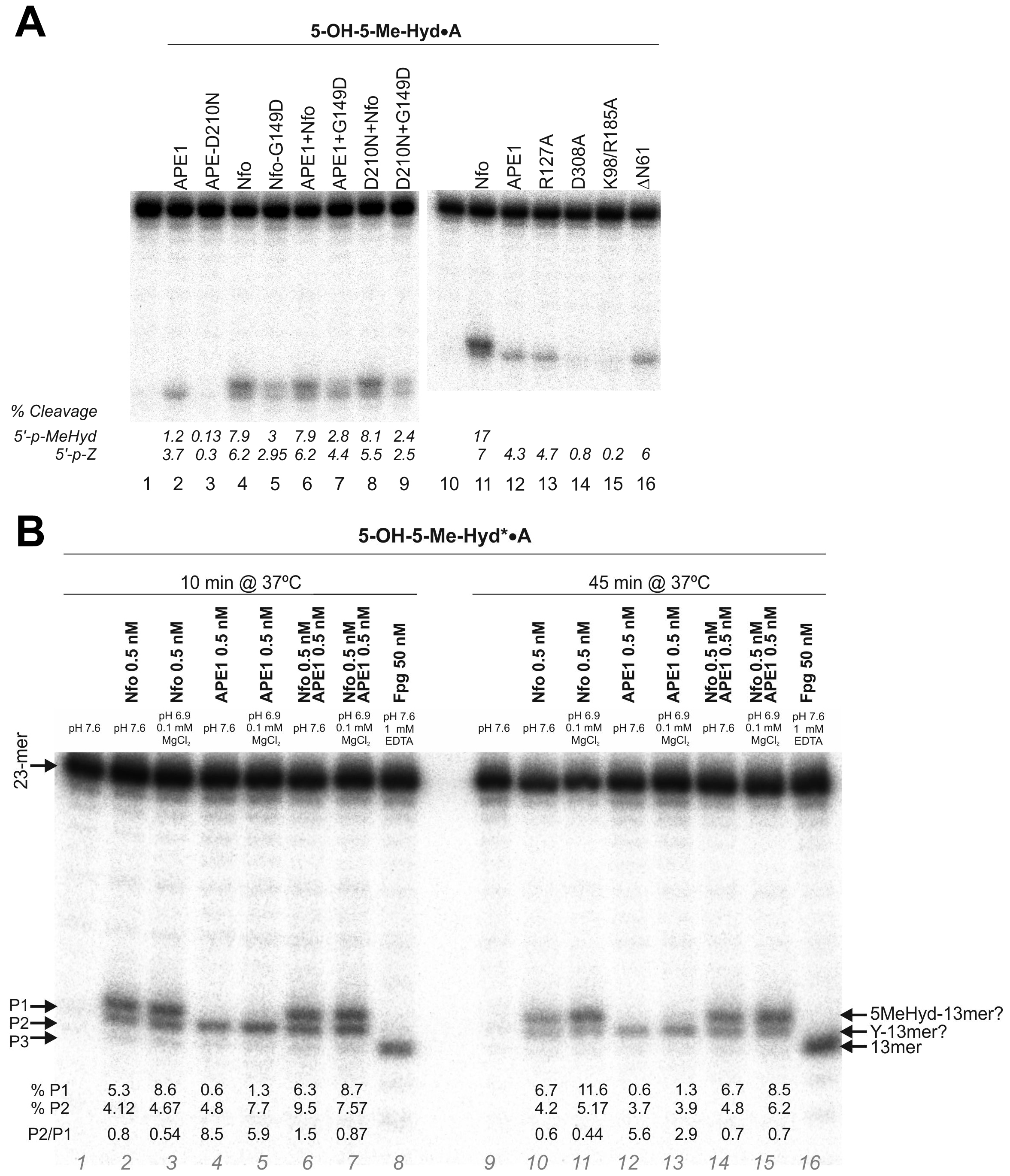

Supplement: Figure S1 — The AP endonuclease activities towards 5OH-5Me-Hyd•A oligonucleotide duplex. The 3′-[32P]-labelled 5OH-5Me-Hyd•A oligonucleotide duplex was incubated with purified WT and mutant Nfo and APE1 under either standard condition “pH 7.6” for Nfo, or “NIR+Mg2+” same as “pH 6.9, 0.1 mM MgCl2” for APE1, or standard condition “BER+EDTA” same as “pH 7.6, 1 mM EDTA” for Fpg. (A) Denaturing PAGE analysis of the cleavage products by WT and mutant AP endonucleases. Nfo-G149G is a highly NIR-deficient mutant of Nfo; R177A and K98E are APE1 mutants with a slightly reduced NIR activity; D308A is APE1 mutant with highly reduced NIR activity; K98A/R185A is APE1 double mutant with highly reduced NIR activity; ΔN61 is a truncated APE1 mutant lacking the N-terminal 61 residues with a slightly reduced NIR activity; D210N is a catalytically inactive APE1 mutant. “5′-p-MeHyd” denotes 14-mer cleavage fragment containing 5′-terminal 5OH-5Me-Hyd residue; “5′-p-Z” denotes 14-mer cleavage fragment containing 5′-terminal ureido residue. (B) Cleavage of 5OH-5Me-Hyd•A oligonucleotide duplex by Nfo, APE1 and Fpg after 10 and 45 min incubation. “P1” and “5MeHyd-13mer” denotes 14-mer cleavage fragment containing 5′-terminal 5OH-5Me-Hyd residue; “P2” and Y-13mer” denotes 14-mer cleavage fragment containing 5′-terminal ureido residue; “P3” and “13mer” denotes 13-mer cleavage fragment containing 5′-phosphate residue. For details see Materials and Methods. (JPG) [file pone.0021039.s001.jpg]

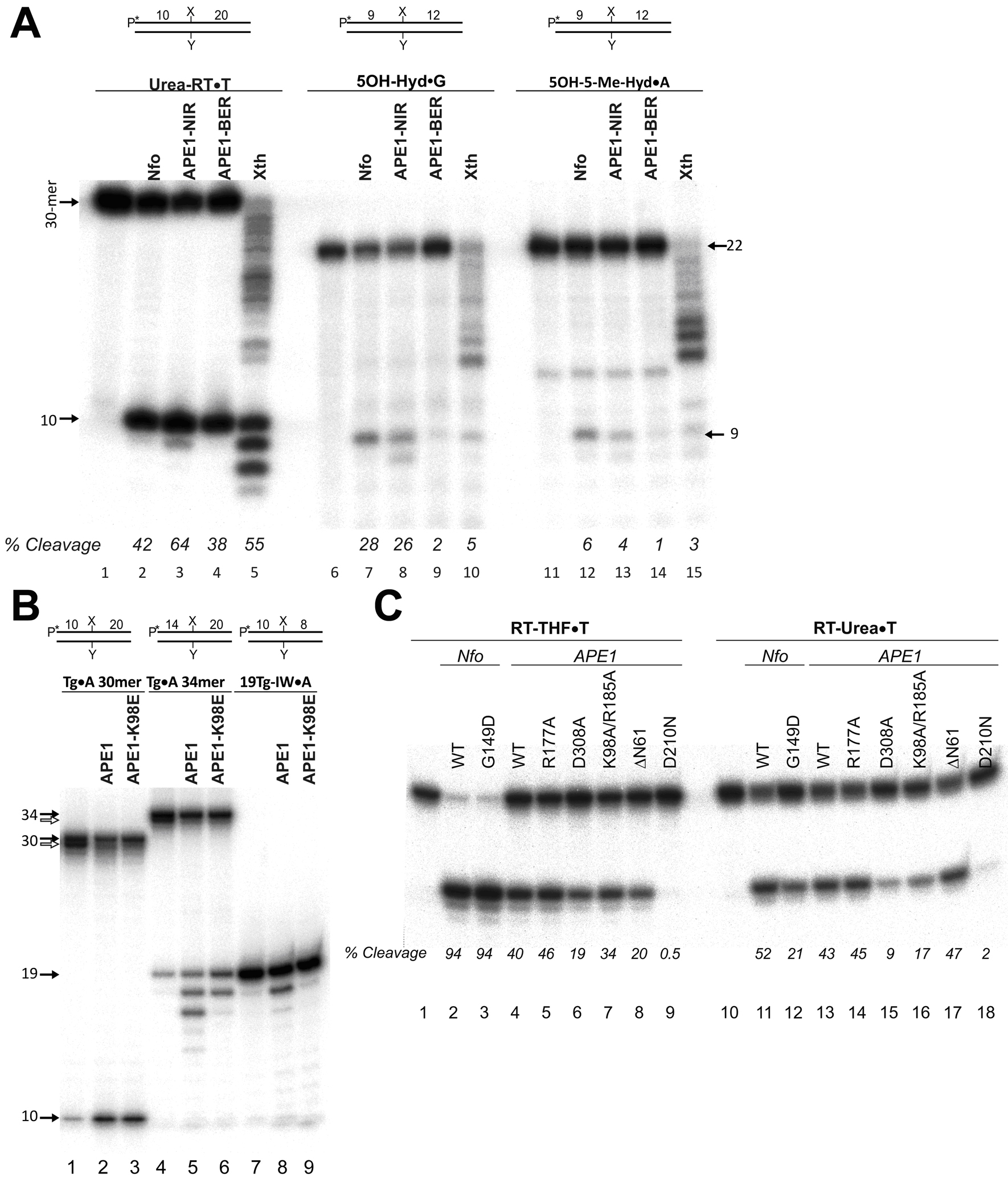

Supplement: Figure S2 — Comparison of the AP endonuclease activities towards urea, hydantoins and thymine glycol containing substrates. 5′-[32P]-labelled oligonucleotide duplexes were incubated with the purified WT and mutant Nfo and APE1 AP endonucleases under standard BER and NIR conditions. Nfo-G149G is a highly NIR-deficient mutant of Nfo; R177A and K98E are APE1 mutants with a slightly reduced NIR activity; D308A is APE1 mutant with highly reduced NIR activity; K98A/R185A is APE1 double mutant with highly reduced NIR activity; ΔN61 is a truncated APE1 mutant lacking the N-terminal 61 residues with a slightly reduced NIR activity; D210N is a catalytically inactive APE1 mutant. (A) Denaturing PAGE analysis of the cleavage products of Urea-RT•T, 5OH-Hyd•G and 5OH-5Me-Hyd•A oligonucleotide duplexes. Lane 1, control non-treated Urea-RT•T; lane 2, as 1 but with Nfo; lane 3, as 1 but with APE1 under NIR condition; lane 4, as 1 but with APE1 under BER condition; lane 5, as 1 but with Xth; lanes 6–10, same as 1–5 but with 5OH-Hyd•G as a substrate; lanes 11–15, same as 1–5 but with 5OH-5Me-Hyd•A as a substrate. (B) Denaturing PAGE analysis of the APE1-cleavage products of 5′-[32P]-labelled 30-mer, 34-mer and 19-mer Tg•A, oligonucleotide duplexes under NIR condition. Following reaction buffers were used for the AP endonucleases: buffer for Nfo contained 20 mM HEPES-KOH, pH 7.6, 50 mM KCl, 0.1 mg/mL BSA and 1 mM DTT treatment; buffer for APE1-NIR contained 20 mM HEPES-KOH, pH 6.9, 50 mM KCl, 0.1 mg/mL BSA, 1 mM DTT and 0.1 mM MgCl2; buffer for APE1-BER contained 20 mM HEPES-KOH, pH 7.6, 100 mM KCl, 0.1 mg/mL BSA, 1 mM DTT and 5 mM MgCl2. Reaction buffer for Xth contained 20 mM HEPES-KOH, pH 7.6, 100 mM KCl, 0.1 mg/mL BSA, 1 mM DTT and 5 mM CaCl2. Lane 1, control non-treated 30-mer Tg•A; lane 2, as 1 but with WT APE1; lane 3, as 1 but with APE1-K98E mutant; lane 4, control non-treated 34-mer Tg•A; lane 5, as 4 but with WT APE1; lane 6, as 4 but with APE1-K98E mutant; lane 7, control non-treated 19 [file pone.0021039.s002.jpg]

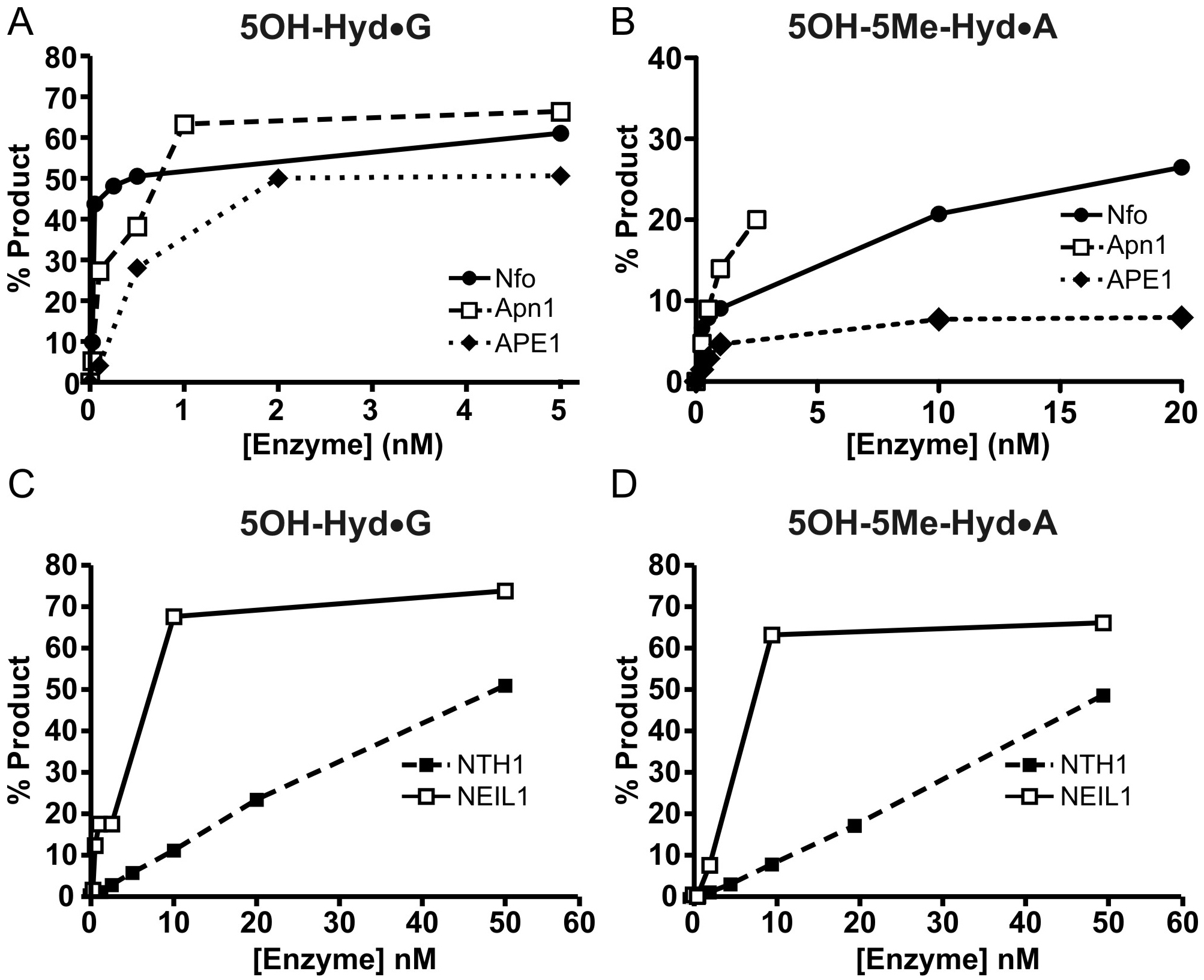

Supplement: Figure S3 — Comparison of the AP endonucleases and DNA glycosylases activities towards pyrimidine-derived hydantoins. Enzyme concentration dependence activity curves on 5′-[32P]-labelled oligonucleotide duplexes containing hydantoins (A) Nfo, Apn1 and APE1 acting on 5OH-Hyd•G; (B) Nfo, Apn1 and APE1 acting on 5OH-5Me-Hyd•A; (C) NTH1 and NEIL1 acting on 5OH-Hyd•G; (D) NTH1 and NEIL1 acting on 5OH-5Me-Hyd•A. For details see Materials and Methods. (JPG) [file pone.0021039.s003.jpg]

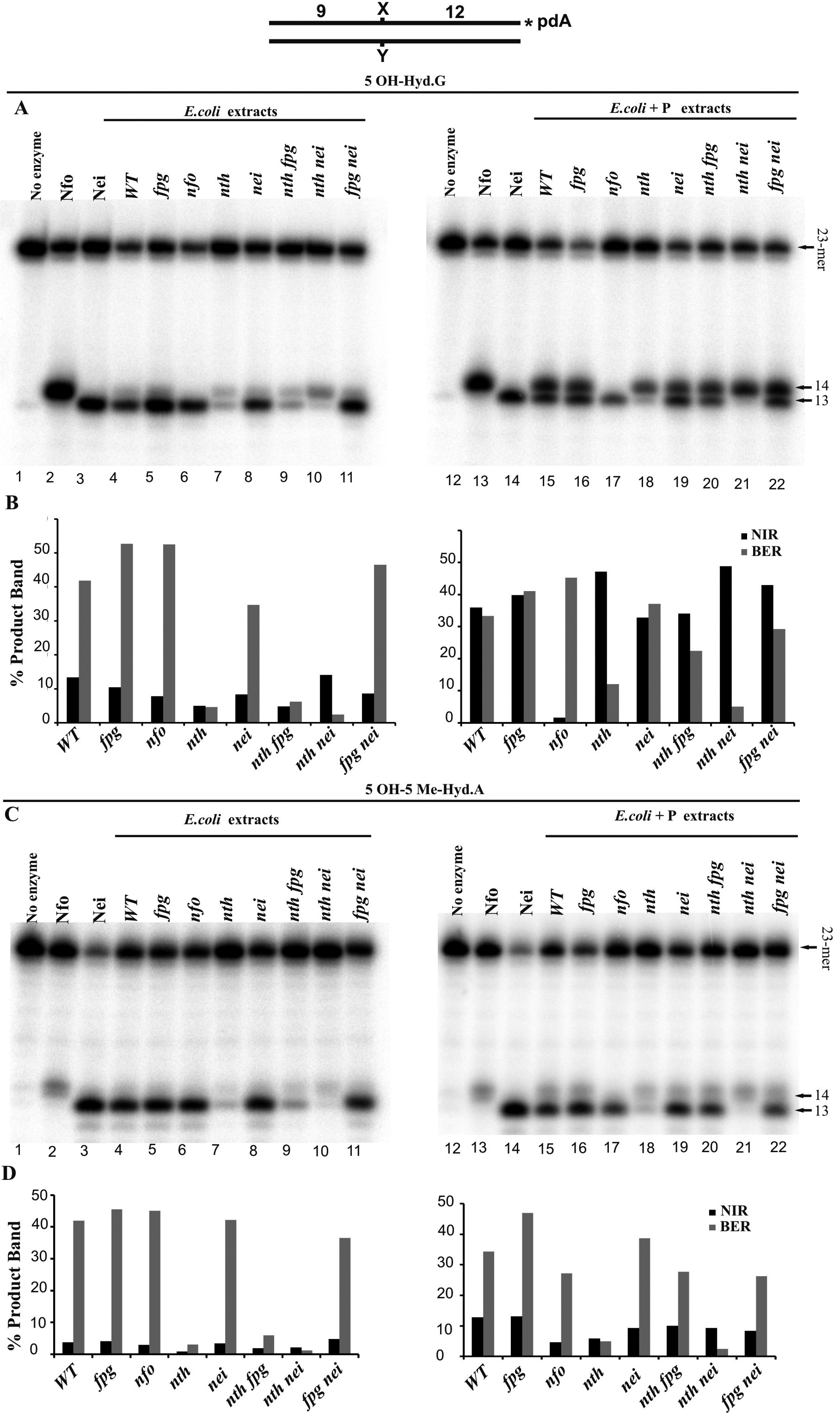

Supplement: Figure S4 — DNA repair activities towards 5OH-Hyd•G duplex in paraquat-induced E. coli cell-free extracts. 3′-[α-32P]-ddATP-labelled oligonucleotide duplexes were incubated with either 3 µg of cell-free extract or limited amount of a purified protein in the standard DNA glycosylase reaction “BER+EDTA” buffer for 30 min at 37°C. (A) Denaturing PAGE analysis of the reaction products of 5OH-Hyd•G oligonucleotide. Lanes 1 and 12, control 5OH-Hyd•G with no enzyme; lanes 2–3 and 13–14, 5OH-Hyd•G incubated with the purified proteins; lanes 4–11, 5OH-Hyd•G incubated with control non-induced extracts; lanes 15–22, 5OH-Hyd•G incubated with paraquat-induced extracts. (B) Graphic representation of the mean values of DNA repair activities on 5OH-Hyd•G. (C) Denaturing PAGE analysis of the reaction products of 5OH-5Me-Hyd•G oligonucleotide. Lanes 1 and 12, control 5OH-5Me-Hyd•A with no enzyme; lanes 2–3 and 13–14, 5OH-5Me-Hyd•A incubated with the purified proteins; lanes 4–11, 5OH-5Me-Hyd•A incubated with control non-induced extracts; lanes 15–22, 5OH-5Me-Hyd•A incubated with paraquat-induced extracts. (D) Graphic representation of the mean values of DNA repair activities on 5OH-5Me-Hyd•A. DNA glycosylase (BER) and AP endonuclease-catalyzed (NIR) incisions were calculated by measuring amount of 13-mer and 14-mer products, respectively. The background values representing control oligonucleotides degradation in absence of enzyme in lanes 1 and 12 were subtracted. “E. coli extracts+P” indicates that the expression of Nfo was induced by exposure of cell culture to 0.25 mg/mL of paraquat. For details see Materials and Methods. (JPG) [file pone.0021039.s004.jpg]

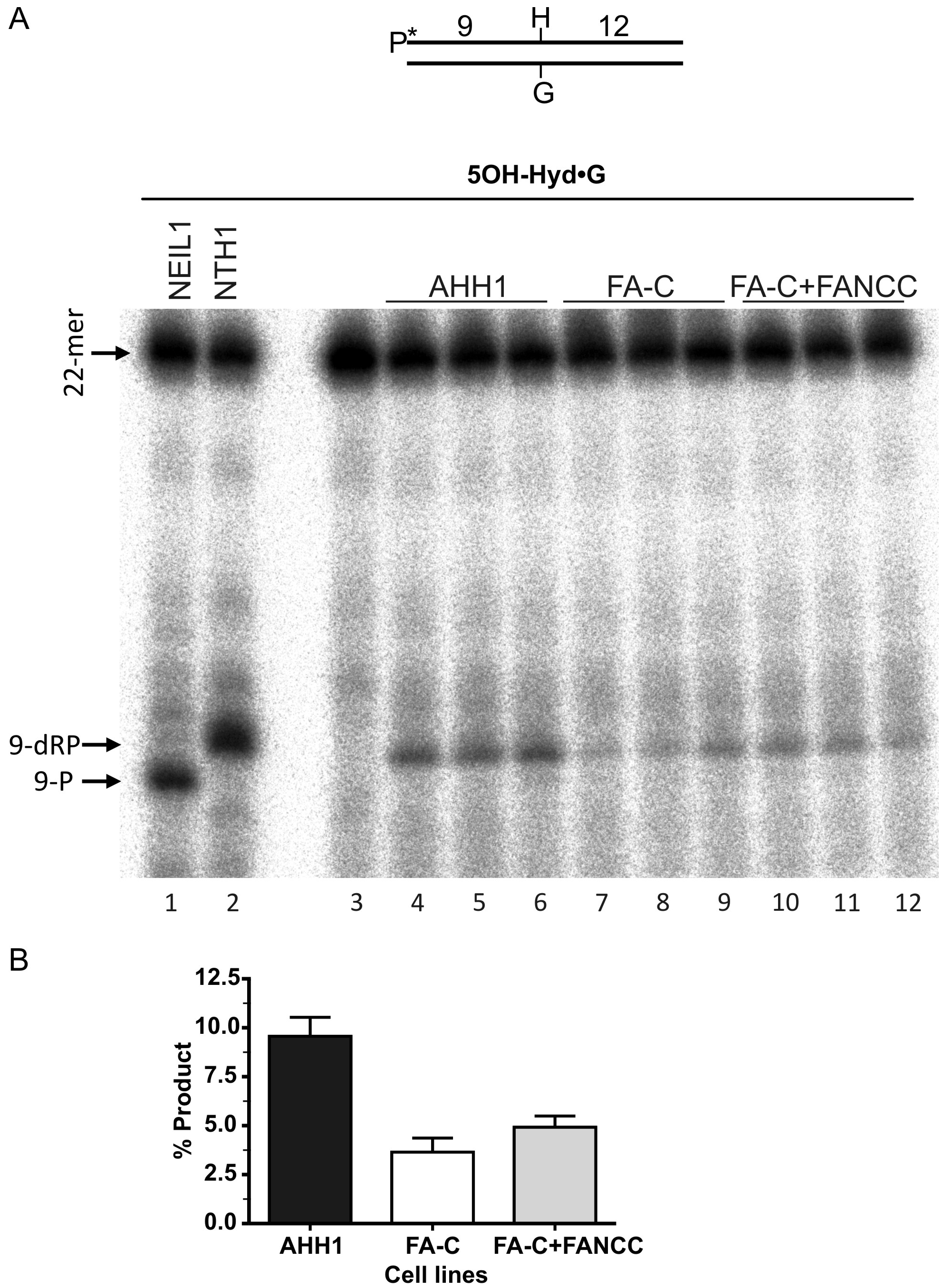

Supplement: Figure S5 — DNA repair activities towards 5-OH-Hyd residues in the extracts of Fanconi complementation group C cells. 5′-[32P]-labelled 5OH-Hyd•G oligonucleotide duplex was incubated with 20 µg of the extracts prepared from AHH1, FA-C and FA-C+FANCC cells. (A) Denaturing PAGE analysis of the reaction products. Lane 1, 5OH-Hyd•G treated with NEIL1; lane 2, 5OH-Hyd•G treated with NTH1; lane 3, control 5OH-Hyd•G no treatment; lanes 4–6, 5OH-Hyd•G treated with AHH1 extracts; lanes 7–9, 5OH-Hyd•G treated with FA-C extracts; lanes 10–12, 5OH-Hyd•G treated with FA-C+FANCC extracts. The arrows denote the position of the 22-mer, 9-mer fragment with 3′-dRP residue (9-dRP) and 9-mer fragment with 3′-phosphate residue (9-P). (B) Graphic representation of the mean values of DNA repair activities in extracts. For details see Materials and Methods. (JPG) [file pone.0021039.s005.jpg]
